# Correlation scan: identifying genomic regions that affect genetic correlations applied to fertility traits

**DOI:** 10.1186/s12864-022-08898-7

**Published:** 2022-10-05

**Authors:** Babatunde S. Olasege, Laercio R. Porto-Neto, Muhammad S. Tahir, Gabriela C. Gouveia, Angela Cánovas, Ben J. Hayes, Marina R. S. Fortes

**Affiliations:** 1 The University of Queensland, School of Chemistry and Molecular Biosciences, Saint Lucia Campus, Brisbane, QLD 4072 Australia; 2 CSIRO Agriculture and Food, Saint Lucia, QLD 4067 Australia; 3 Animal Science Department, Veterinary School, Federal University of Minas Gerais, Belo Horizonte, 31270-901 Brazil; 4 Department of Animal Biosciences, Centre for Genetic Improvement of Livestock, University of Guelph, 50 Stone Rd E, Guelph, ON N1G 2W1 Canada; 5 The University of Queensland, Queensland Alliance for Agriculture and Food Innovation (QAAFI), Saint Lucia Campus, Brisbane, QLD 4072 Australia

**Keywords:** Genomic correlation, Drivers, Antagonizing, RHOGDI, Pathway analysis, QTLs, Correlation scan

## Abstract

Although the genetic correlations between complex traits have been estimated for more than a century, only recently we have started to map and understand the precise localization of the genomic region(s) that underpin these correlations. Reproductive traits are often genetically correlated. Yet, we don’t fully understand the complexities, synergism, or trade-offs between male and female fertility. In this study, we used reproductive traits in two cattle populations (Brahman; BB, Tropical Composite; TC) to develop a novel framework termed correlation scan (CS). This framework was used to identify local regions associated with the genetic correlations between male and female fertility traits. Animals were genotyped with bovine high-density single nucleotide polymorphisms (SNPs) chip assay. The data used consisted of ~1000 individual records measured through frequent ovarian scanning for age at first *corpus luteum* (AGECL) and a laboratory assay for serum levels of insulin growth hormone (IGF1 measured in bulls, IGF1b, or cows, IGF1c). The methodology developed herein used correlations of 500-SNP effects in a 100-SNPs sliding window in each chromosome to identify local genomic regions that either drive or antagonize the genetic correlations between traits. We used Fisher’s Z-statistics through a permutation method to confirm which regions of the genome harboured significant correlations. About 30% of the total genomic regions were identified as driving and antagonizing genetic correlations between male and female fertility traits in the two populations. These regions confirmed the polygenic nature of the traits being studied and pointed to genes of interest. For BB, the most important chromosome in terms of local regions is often located on bovine chromosome (BTA) 14. However, the important regions are spread across few different BTA’s in TC. Quantitative trait loci (QTLs) and functional enrichment analysis revealed many significant windows co-localized with known QTLs related to milk production and fertility traits, especially puberty. In general, the enriched reproductive QTLs driving the genetic correlations between male and female fertility are the same for both cattle populations, while the antagonizing regions were population specific. Moreover, most of the antagonizing regions were mapped to chromosome X. These results suggest regions of chromosome X for further investigation into the trade-offs between male and female fertility. We compared the CS with two other recently proposed methods that map local genomic correlations. Some genomic regions were significant across methods. Yet, many significant regions identified with the CS were overlooked by other methods.

**Supplementary Information:**

The online version contains supplementary material available at 10.1186/s12864-022-08898-7.

## Background

In animal genetics, insight into the nature of the genetic relationships between quantitative traits are important because they improve our understanding of complex traits and diseases [1, 2]. These relationships termed genetic correlations manifest when there is shared genetic influence between traits (i.e., pleiotropy) [3, 4] or when there is non-random association between loci (i.e., linkage disequilibrium (LD)) [5, 6]. Estimated genetic correlations provide information on how genome-wide genetic effects align between two complex traits [7]. Understanding the interplay between genomic variants and their effects on quantitative traits can yield insights to improve the prediction of genetic merit and the understanding of complex traits’ biology [8–10]. Estimated genetic correlations have informed animal and crop breeding for many decades. For example, scrotal circumference is used as an indicator trait in beef cattle breeding because it is genetically correlated with female fertility traits [11]. Nevertheless, we still have a limited information of the genomic regions regulating the intersexual correlations between male and female fertility traits. Investigating these regions and leveraging on the resulting biological information could inspire new approaches in livestock breeding [12, 13].

Over the past 100 years, different methods have been employed to estimate the genetic correlation between traits [14–17]. Traditionally, these correlations are estimated from pedigree data. However, genome-wide single nucleotide polymorphisms (SNPs) are often used in recent times [18]. It is possible to estimate across-sex correlation between traits and this research niche continues to attract interest among quantitative geneticists [19–21]. The resulting estimates from both within and across-sex analyses range from − 1 to + 1, indicating the strength and magnitude of the correlation between traits [22]. Despite more than a century of research on estimating this parameter, it is only very recently that studies attempt to identify local regions that underpin genetic correlations between traits [23, 24, 25]. For instance, in human genetics, methods such as ρ-HESS [23], SUPERGNVOVA [25], and LAVA [24] have been developed for this purpose. However, in livestock genetics, there is currently no methodology aim to precisely estimate and identify these local regions. For human genetics, local correlations between traits are usually estimated using genome-wide association studies (GWAS) summary statistics with different model assumptions. To account for LD in local correlation estimates, these methods used external genotype data by assuming that the LD structures are identical to the GWAS summary data. Sawyer et al. [26] showed that the pattern of LD can vary substantially, even for population in the same geographic location. The imperfect LD structure between the external genotype data and GWAS summary data can create estimation uncertainty and leads to false positive or negative results in the local correlation estimates [27]. In addition to this limitation, some estimates of local correlations from these methodologies are often unstable due to noise in the local heritability estimates, resulting in local estimates that are often out of bound [24, 25]. As a result, these local estimates are either capped at +/− 1 or excluded from the analyses. Therefore, there is need to develop a method that can estimate local genomic correlation without relying on local heritabilities and can also account for the pattern of LD using the population under study. Here, we introduce correlation scan (CS), a simple, fast, and robust method that uses sliding window approach to identify local genomic regions affecting trait correlation using post-GBLUP (genomic best linear unbiased prediction) SNP effects.

Furthermore, local genomic correlation can either drive a global correlation estimate or antagonize it. In theory, various genomic regions will contribute to the global correlation between complex traits. Further, some regions will be driving (i.e., driver regions) the global genetic correlation while others might antagonize it (antagonizing regions). For instance, if the global genetic correlation between two traits is 0.70, some local regions will yield a significant and positive correlation, say 0.90, while other regions may antagonize the overall estimate, and in that region the correlation could be − 0.50. Also, some local genomic regions may be neutral, say 0.02 and not significant for the correlation between the studied traits. Identifying driver and antagonizing regions are of particular interest if they are for two important traits which are unfavourably correlated, for example milk yield and fertility in dairy cattle. Identification of such regions could lead to more targeted genomic selection and rapid genetic gains for both traits. Current genomic tools have created a great opportunity to advance our knowledge of genetic correlations between complex traits, by investigating the regions in the genome that drive or antagonize these correlations.

Here, we applied the CS method to real datasets, i.e., male and female fertility traits to identify drivers and antagonizing regions affecting these traits. These identified drivers and antagonizing regions were subjected to QTLs enrichment and functional analysis to gain insight about the biology of the traits under study. The traits used are age at first *corpus luteum* (AGECL, i.e., female puberty) and serum levels of insulin growth hormone (IGF1 measured in bulls, IGF1b, or cows, IGF1c). These phenotypes are from animals raised in a research station with precise measurement taken by regular ovarian scan (AGECL, [26]) and laboratory assay (IGF1c or IGF1b, [27, 28]). These traits are highly heritable [28, 29] and each pair serve as example of a positive and a negative correlation between phenotypes measured in males and females, during pubertal development. The populations used in the study are formed by either Brahman (BB) cattle or Tropical Composite (TC) cattle, as described in our previous study [29]. For completeness, we compared the CS methodology with two other recently proposed methods (SUPERGNOVA and LAVA) that map local genomic correlations.

## Results

### The total number of windows generated

**Fig. 1:**
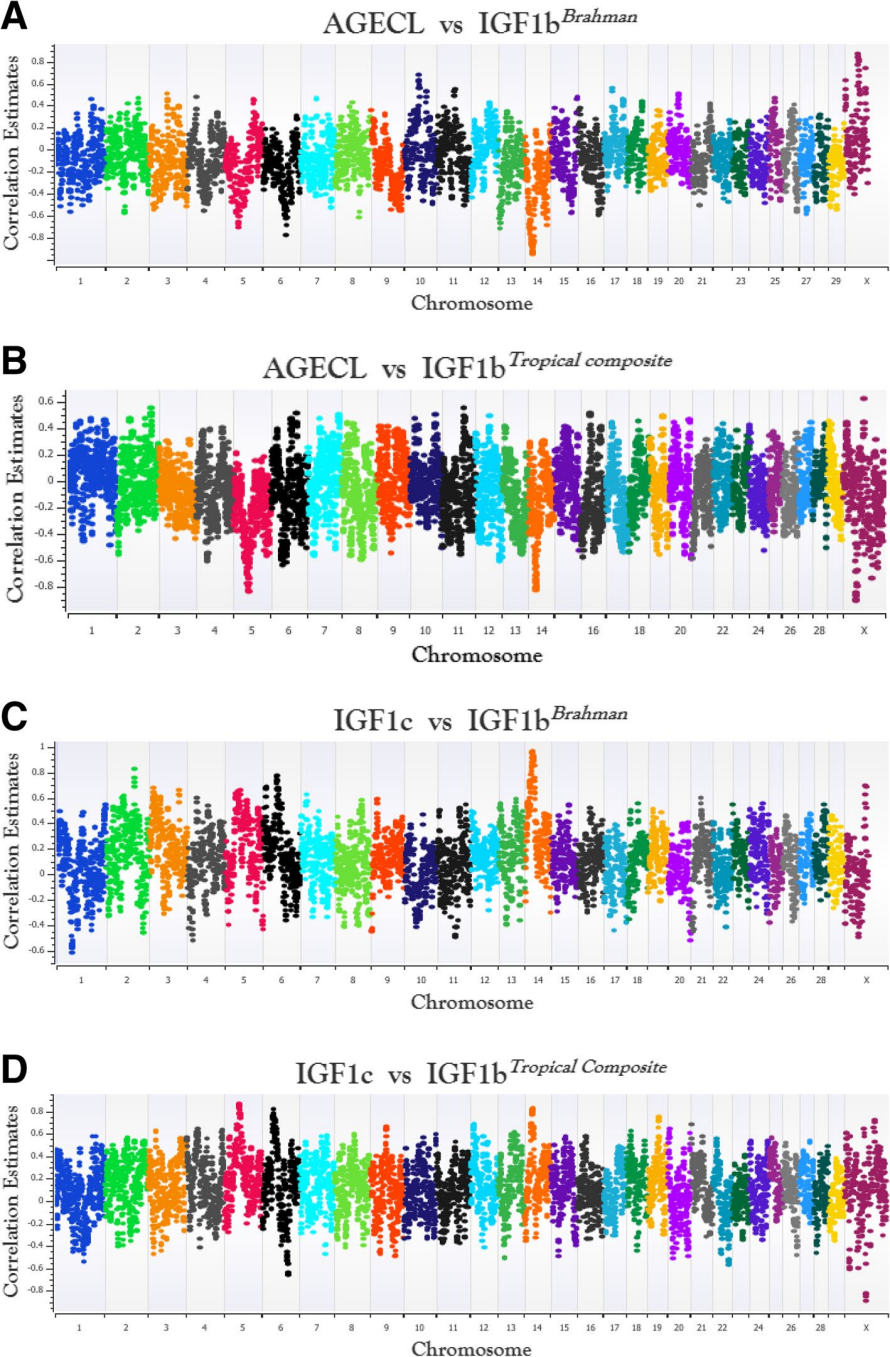
Genome-wide plot of the sliding window local correlation estimates for the trait pairs (BB-AGECL vs IGF1b; **A**, TC-AGECL vs IGF1b; **B**, BB-IGF1c vs IGF1b; **C**, and TC- IGF1c vs IGF1b; **D**) in Brahman (BB) and Tropical Composite (TC). AGECL, age at first *corpus* *luteum*; IGF1, serum levels of insulin growth hormone (measured in bulls, IGF1b, or cows, IGF1c). The correlation estimates were plotted on the y-axis and the genomic position (i.e., midpoint between the start and end position of each window) of each chromosome on the x-axis, according to the ARS_UCD1.2 bovine reference genome using SNP & Variation Suite v8.x Golden Helix [30]. Only **r** estimates with Bonferroni-corrected *P*-value < 0.05 were considered significant

Using the framework developed in our study (see Materials and Methods), genomic windows with their corresponding local genetic correlation (**r**) estimates for each pairwise trait in two beef cattle populations were identified. The total number of windows generated for all trait pairs in BB was 5413 and the number in TC was 6731. The **r** estimates for all windows were plotted against their genomic position (i.e., midpoint between the start and end position of each window) (Fig. 1). Results are presented separately per cattle population and for each pair of traits investigated. Additionally, the full details of these results are presented in Additional file 1 (Table S1-S2).

In addition, to demonstrate that the SNP effects from the back-solve approach using GBLUP are not biased by LD, we pruned the genotypes data of each breed based on pairwise LD estimates to obtain a pruned subset of SNPs that are in approximate linkage equilibrium (LE). Compared to the genotype data (~ 600 K SNPs for each breed), about 164 k and 194 K SNPs remained after LD pruning for BB and TC breed, respectively. In summary, we observed no substantial differences between the pattern of the **r** estimates for the LD pruned SNP effects and those of the back-solve approach using GBLUP (Additional file 2; Fig. S1).

### Identification of significant windows

To identify windows with significant **r** estimates, we applied Fisher’s Z-statistics following a permutation test of 10,000 iterations (see Methods). Significant level was set at Bonferroni-corrected *P*-value < 0.05. Windows with** r** estimates that passed this threshold were considered significant and those that failed were termed “neutral” windows. Table 1 shows the number of significant and neutral windows for each pair of trait in each population. About 30% of the total genomic regions were significant for each trait pair. The higher the global correlation between trait pairs, the higher the number of significant windows and the lesser the neutral windows.

**Table 1:** The number of significant (*P*-value < 0.05) and neutral (*P*-value> 0.05) windows for each trait pair in the Brahman and Tropical Composite population

Trait pair	Number of windows (percentage in parenthesis)		Total number of windows
	Significant windows (%)	Neutral (%)	
*Brahman*			
AGECL vs IGF1b	1305 (24.11%)	4108 (75.89%)	5413
IGF1c vs IGF1b	1518 (28.04%)	3225 (71.96%)	5413
*Tropical Composite*			
AGECL vs IGF1b	1731 (25.72%)	5000 (74.28%)	6731
IGF1c vs IGF1b	2103 (31.24%)	4628 (68.76%)	6731

*AGECL* age at first *corpus*, *IGF1c* serum levels of insulin growth hormone measured in cow, *IGF1b* serum levels of insulin growth hormone measured in bulls

### Comparison of CS with SUPERNOVA and LAVA

To demonstrate the performance of CS, we compared the results from CS with SUPERNOVA and LAVA across all the trait pairs investigated. Additional file 3 (Table S3-S10) shows the **r** estimates for each local region (window) and their corresponding *P*-values for SUPERGNOVA and LAVA. Of note, the **r** estimated in SUPERGNOVA depends on local heritability estimates, and in many cases, these estimates are unstable and often negative. SUPERGNOVA ignores negative heritability estimates, leaving the correlation estimates as ‘NA’. Furthermore, some of the estimates are also out of bound, i.e., estimates with **r** > 1 or **r** < − 1. LAVA, on the other hand, tends to avoid unstable local correlation estimates by filtering out non-associated loci with low local heritability. Despite this, some estimates are often out of bounds and are either capped at +/− 1 (i.e., if r estimate is <+/− 1.25) or excluded from the analyses (i.e., if r estimate is >+/− 1.25). In addition, the **r** estimate in LAVA depends on variance of the phenotypes. If the variance of local region is negative, the window will be excluded from the analysis.

For SUPERGNOVA, about 5%, on average, of the total number of windows analysed were within the **r** range (i.e., − 1 ≤ r ≤ 1) for all traits pairwise (Table 2). Approximately 85% of the total windows were NA due unstable local heritability estimate and about 10% were out of bound (**r** > 1).

**Table 2:** The proportion of unstable local estimates (NA’s), out of bound estimates (**r** > 1) and within bound estimates (i.e., − 1 ≤ **r** ≤ 1) to the number of windows analysed for all traits pairwise for SUPERGNOVA in Brahman and Tropical Composite population

Trait pairs	No of windows analysed	NA’s	Out of bound estimates	Within bound estimates
*Brahman*				
AGECL vs IGF1b	5413	87%	10%	3%
IGF1c vs IGF1b	5413	85%	10%	5%
*Tropical Composite*				
AGECL vs IGF1b	6731	83%	11%	6%
IGF1c vs IGF1b	6731	83%	12%	5%

For LAVA, out of the total number of windows (BB, 5413; TC, 6731), about 30 and 60% of the total windows were analysed for all trait pairs in BB and TC, respectively (Table 3). The rest were excluded from the analyses due to negative variance of either one or both phenotypes being investigated. Of those analysed, about 11-30% had their **r** estimate either capped at +/− 1 or out of bound.

**Table 3:** The number of windows analysed and excluded for all trait pairs for LAVA in Brahman and Tropical Composite population. Proportion of estimate capped at +/−1, out of bound estimate (NA’s) and within bound estimates (i.e., − 1 ≤** r** ≤ 1) are relative to the number of windows analysed

Trait pairs	No of windows	No of windows excluded	No of window analysed	Estimate capped at +/−1	Out of bound estimates	Within bound estimates
*Brahman*						
AGECL vs IGF1b	5413	3733 (69%)	1680 (31%)	8%	25%	67%
IGF1c vs IGF1b	5413	3640 (67%)	1773 (33%)	3%	8%	89%
*Tropical Composite*						
AGECL vs IGF1b	6731	2903 (43%)	3828 (57%)	6%	16%	78%
IGF1c vs IGF1b	6731	2650 (39%)	4081 (61%)	8%	23%	69%

**Fig. 2:**
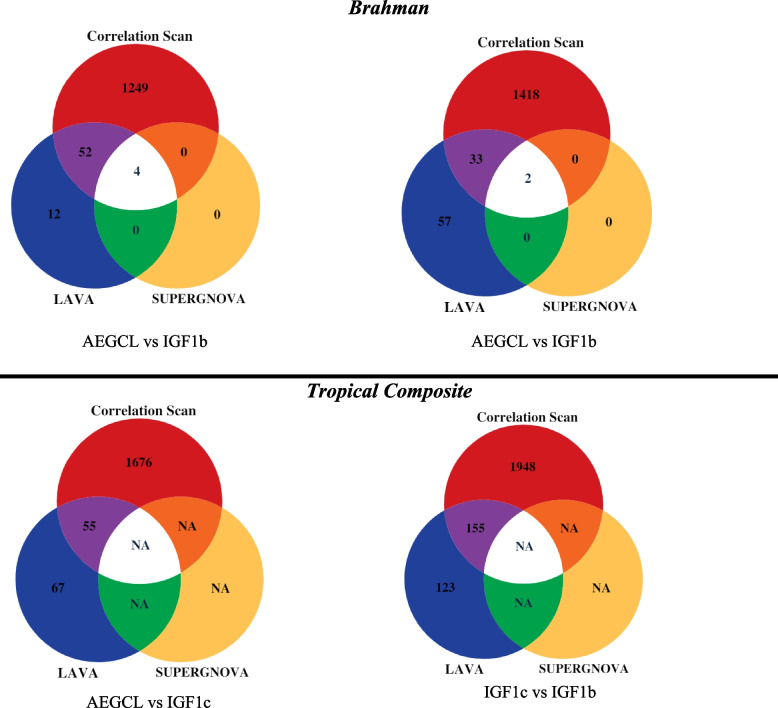
The Venn Diagram showing the number of significant local genetic correlations detected using correlation scan, SUPERGNOVA and LAVA at Bonferroni-corrected *P*-value < 0.05 for all trait pairs in the Brahman (top) and Tropical Composite (bottom) populations

We also investigated the number of significant windows shared between CS with SUPERGNOVA and LAVA for all trait pairs in the BB and TC population at *P*-value < 0.05 (Fig. 2). For SUPERGNOVA, 4 windows were significant for AGECL vs IGF1b and 2 windows were significant for IGF1c vs IGF1b in BB population. No window was significant for any of the trait pairs in TC population. The significant windows for the two pairwise traits in BB overlapped with the significant windows detected using CS. These overlaps were on the bovine chromosome (BTA) 14: 22.68-25.29 Mb and corresponded with the regions with the largest and the most significant **r** estimate with CS.

For LAVA, 68 windows were significant for AGECL vs IGF1b and 92 for IGF1c vs IGF1b at *P*-value < 0.05 in BB. The number of overlaps with CS for these windows were 56 and 92 for AGECL vs IGF1b and IGF1c vs IGF1b, respectively. In TC, 122 windows were significant for AGECL vs IGF1b and 278 for IGF1c vs IGF1b at *P*-value < 0.05. The number of overlaps with CS for these windows were 55 and 155 for AGECL vs IGF1b and IGF1c vs IGF1b, respectively.

**Fig. 3:**
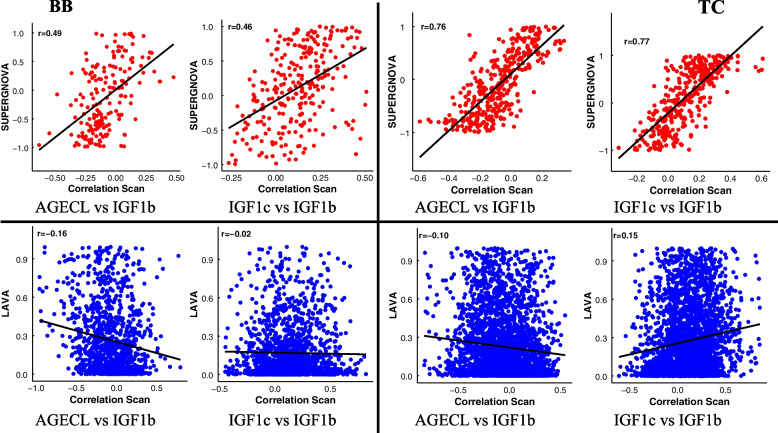
Relationship between the estimated local genetic correlations obtained from SUPERGNOVA (top) and LAVA (bottom) with correlation scan in BB (left) and TC (right). These plots exclude NA’s and out of bounds values from SUPERGNOVA and LAVA. AGECL, age at first corpus; IGF1b, serum levels of insulin growth hormone measured in bulls

Furthermore, we investigated the relationship between the **r** estimates (i.e., within bound estimates only) from CS with SUPERGNOVA and LAVA in the two population for all trait pairs studied (Fig. 3). For SUPERGNOVA, we observed moderate correlations with CS for AGECL vs IGF1b (0.49) and IGF1c vs IGF1b (0.46) in BB population. However, in TC, we observed high correlations with correlations scan (~ 0.76) for these two traits pair. For LAVA, the relationship of the **r** with CS for all trait pairs were low and the directions were negative except for IGF1c vs IGF1b in TC population.

### Driver and antagonizing windows affecting genetic correlations between fertility traits

The significant **r** estimates facilitated the identification of driver and antagonizing windows. Depending on the global correlation between traits, driver and antagonizing windows can be deduced: in driver windows, the **r** estimate has the same direction, positive or negative, as the global genetic correlation; in antagonizing windows it is the opposite. Additional file 4 (Table S11) shows the number of significant drivers and antagonizing windows for all trait pairs in BB and TC populations.

The number of significant driver windows for the correlation between AGECL and IGF1b was 1022 in BB and 1230 in TC cattle. The number of significant windows for the antagonizing windows was 283 in BB and 501 in TC cattle, for AGECL vs IGF1b. For the correlation between IGF1c and IGF1b, the number of significant driver windows was 1293 in BB and 1765 in TC cattle. The antagonizing windows was 225 in BB and 338 in TC cattle (IGF1c vs IGF1b). In addition, the lists of windows with their chromosome position and genomic coordinates for all driver, and antagonizing regions are presented in Additional file 5 (Table S12-S19).

For the correlation between AGECL and IGF1b (global genetic correlation of − 0.65 (BB) and − 0.55 (TC), see Additional file 6; Table S20), the largest **r** estimate for the driver windows was − 0.96 (bovine chromosome (BTA)14: 23.04 - 25.29 Mb) in BB and − 0.91 (BTAX: 39.76 - 42.86 Mb) in TC. For the antagonizing windows, the largest **r** estimate was 0.87 (BTAX: 40.87 - 43.88 Mb) in BB and 0.62 (BTAX: 66.62 - 69.622 Mb) in TC.

For the correlation between IGF1c and IGF1b (global genetic correlation of 0.86 (BB) and 0.93 (TC), see Table 2), the largest **r** estimate for the driver windows was 0.97 (BTA14: 22.68 - 24.96 Mb) in BB and 0.87 (BTA5: 46.13- 47.89 Mb) in TC, while the estimate for the antagonizing was − 0.62 (BTA1: 49.01 - 51.67 Mb) in BB and − 0.90 (BTAX: 65.64 - 68.39 Mb) in TC. All **r** estimates are plotted in Fig. 2.

### Genes and quantitative trait loci (QTLs) within driver and antagonizing regions across the two populations

**Fig. 4:**
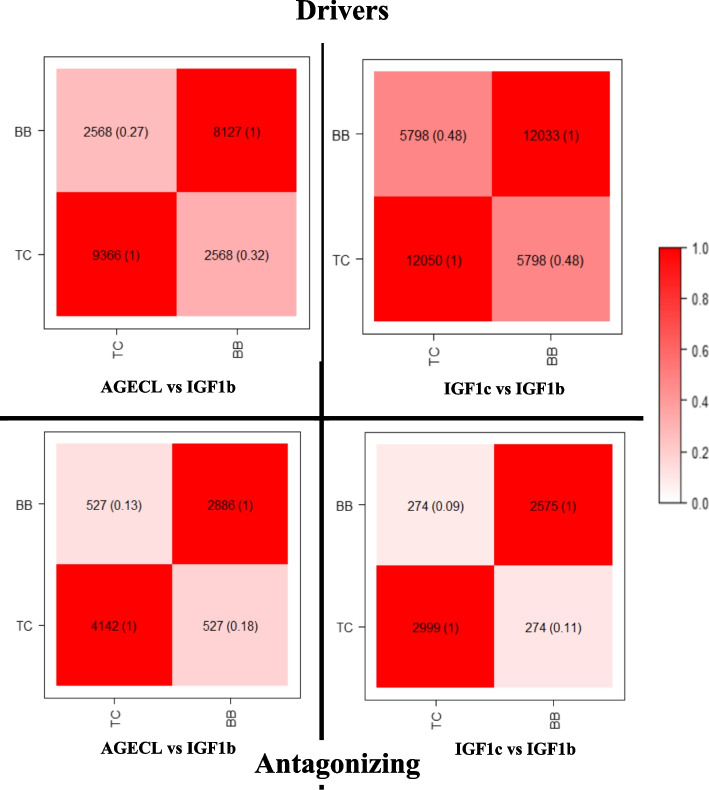
Genes annotated in the significant (i.e., driver and antagonizing) genomic regions identified as explaining the genetic correlations between male and female fertility traits in Brahman (BB) and Tropical Composite (TC) population. The overlaps between the two studied populations are in the diagonal of each plot for each pair of traits within the driver (above) and antagonizing (below) regions. The parenthesis describes the percentages of genes shared across the significant regions in BB and TC calculated as a function of the total number of genes in BB or TC. The darker the colour within the squares, the higher the percentage of shared genes or QTLs. AGECL, age at *first corpus*; IGF1, serum levels of insulin growth hormone (measured in bulls, IGF1b, or cows, IGF1c)

**Fig. 5:**
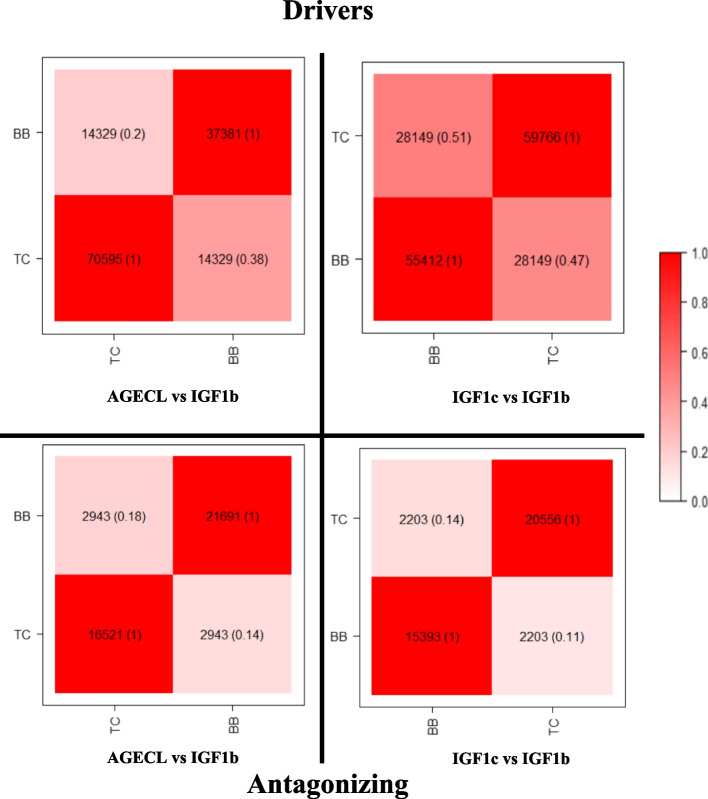
QTLs annotated in the significant (i.e., driver and antagonizing) genomic regions identified as explaining the genetic correlations between male and female fertility traits in Brahman (BB) and Tropical Composite (TC) population. The overlaps between the two studied populations are in the diagonal of each plot for each pair of trait within the driver (above) and antagonizing (below) regions. The parenthesis describes the percentages of genes shared across the significant regions in BB and TC calculated as a function of the total number of genes in BB or TC. The darker the colour within the squares, the higher the percentage of shared genes or QTLs. AGECL, age at first corpus luteum; IGF1, serum levels of insulin growth hormone (measured in bulls, IGF1b, or cows, IGF1c)

Defining driver and antagonizing regions separately for each pair of traits, allowed us to identify the genes and QTLs within these regions for each of the two beef cattle populations. The percentage of the overlapping genes (Fig. 4) and QTLs (Fig. 5) across both populations was studied. The percentages of genes shared across the significant regions in BB and TC were calculated as a function of the total number of genes in BB or TC, respectively, and so they differ (Figs. 4 and 5).

The percentage of overlapping genes for each pair of traits in the two populations were as follows: for AGECL vs IGF1b driver regions, 32% of genes annotated in BB were present in TC and 27% of genes annotated in TC were found in BB, whereas, for the antagonizing regions 18% of genes annotated in BB were present in TC and 13% of genes annotated in TC were found in BB. For IGF1c vs IGF1b, the two populations shared 48% of total number of genes annotated for the driver regions and about 10% were shared for the antagonizing regions.

The percentage of overlapping QTLs for each pair of traits in BB and TC population were as follows: for AGECL vs IGF1b driver regions, 38% of the QTLs annotated in BB were present in TC and 20% of the QTLs annotated in TC were present in BB, whereas, for the antagonizing regions, 14% of the QTLs annotated in BB were present in TC and 18% of the QTLs annotated in TC were present in BB. For IGF1c vs IGF1b, 51% of the genes annotated in BB were present in TC, and 47% of the genes annotated in TC were present in BB, whereas, for the antagonizing regions, 14% of the genes annotated in BB were present in TC and 11% of the genes annotated in TC were present in BB population.

### Functional classification of QTLs within genomic regions that explain the genetic correlations between male and female fertility

To infer biological function and mine the existing literature, we examined the classes of QTL (milk, reproduction, production, meat and carcass, health and exterior) present in the significant genomic regions identified above using GALLO [31]. The largest proportion of QTL classes across all pairwise traits in the two populations for the driver and antagonizing regions were QTLs related to milk production, accounting for about 27-50% in most cases. This was followed by reproductive QTLs accounting for about 13-52% and production QTLs comprising 4-28%. Other QTL types (Exterior, health and meat and carcass) accounted for a relatively small proportion of QTLs in the significant regions (Additional file 7; Fig. S2-S3). In addition, we reported the top 10 results (bar plots) for trait-specific QTLs related to reproduction as these are relevant to our studied traits (Additional file 7; Fig. S2-S3). Among these reproductive QTLs, traits related to puberty (i.e., age at puberty, scrotal circumference) were prevalent in both populations.

### QTL enrichment analysis

We performed a chromosome-wide QTL enrichment analysis to further test the significance of the QTLs identified for all the driver and antagonizing regions in each cattle population, for each trait pair using GALLO [31]. Enriched QTLs for the studied traits span across most QTL types, indicating the presence of complex genetic mechanisms. The results of the chromosome-wide QTLs enrichment (FDR-corrected *P*-value< 0.05) for the driver and antagonizing regions for all pairwise traits in each population are presented in Additional file 8 (Table S21-28).

For the driver regions, the number of QTLs enriched over a wide range of chromosomes for AGECL vs IGF1b were 254 and 157 in BB and TC beef cattle population, respectively. The number was 213 (BB) and 198 (TC) for IGF1c vs IGF1b. For AGECL vs IGF1b, the most enriched chromosome (no of enriched QTLs in parenthesis) was BTA14 (33) and BTA14 (19) in BB and TC, respectively. For IGF1c vs IGF1b, the most enriched chromosome was on BTA5 (43) in BB and BTA14 (46) in TC.

For the antagonizing regions, the number of QTLs enriched across the bovine chromosomes for AGECL vs IGF1b were 102 and 171 in BB and TC beef cattle population, respectively. The number was 136 (BB) and 154 (TC) for IGF1c vs IGF1b. For AGECL vs IGF1b, the most enriched chromosome in BB were both BTA2 (12) and BTA17 (12) in BB, whereas in TC, the most enriched chromosome was BTA26 (29). For IGF1c vs IGF1b, on the other hand, the most enriched chromosome was BTA6 (26) and BTA26 (29) in BB and TC, respectively.

To identify the common results and shared biology between the driver and the antagonizing regions, we also investigated the overlaps of the QTL types associated with the studied trait (i.e., reproduction) in the two populations. The relationship between the top 10 enriched reproductive QTLs in BB and TC are presented in Additional file 9 (Fig. S4). Irrespective of the trait pairs, for the driver regions, the reproductive QTLs in BB in most cases overlap with those identified in TC. However, for the antagonizing regions, not all reproductive QTLs in BB were found in TC beef cattle population.

### Functional enrichment analysis

Leveraging our methodology’s directionality of gene effects with Ingenuity Pathway Analysis (IPA; http://www.ingenuity.com), we identified the canonical metabolic pathways enriched at Benjamini–Hochberg corrected *P*-values (BH-*P*-value) of *p* < 0.01. The graphical presentation of the canonical metabolic pathways predicted by IPA to be enriched and the proportion of driver and antagonizing genes in each pathway for all pairwise traits investigated in each population are illustrated in Additional file 10 (Fig. S5-S12). Although IPA provided information about whether the predicted pathways were being activated or inhibited based on our data, we remain cautious when interpreting our results since the **r** estimates are not the same as gene expression values, and IPA was originally designed to mine gene expression data. In summary, several biological pathways known to be involved in reproduction (i.e., studied trait) were significantly enriched for all pairwise traits investigated across the two breeds. Rho GDP Dissociation Inhibitor (RHOGDI) pathway was the only significant signaling pathway found to be inhibited across breeds in all pairwise traits investigated using IPA comparison analysis.

## Discussion

Complex phenotypes, like fertility, consist of multiple genetically correlated traits rather than independent traits [6]. The correlations between complex traits involve many genomic regions, with genes that could be part of a large and polygenic regulatory network [32–35]. Genomic signals that regulate (i.e., drive or antagonize) the correlations between complex traits are widely spread across the genome. Genomic regions underpinning trait correlation include genes with and without a significant effect on each specific phenotype or disease [33]. In the present post-genomic era, discovering the genomic regions that regulate correlations between complex traits has become an important aspect of genetic studies in humans and animals [36]. In this study, we developed a novel framework termed CS to reveal the significant regions that either drive or antagonize the genetic correlations between traits across the genome. In addition, this method can also reveal genomic regions with no effect on the studied traits (neutral windows). The CS uses SNP effects, from best linear unbiased predictions (BLUP), to estimate the local correlations between studied traits. Local correlations are based on sliding windows of 500-SNPs. We applied this sliding windows approach to reproductive traits measured in two populations of cattle. We subjected the significant windows to further analyses using GALLO [31] and IPA (http://www.ingenuity.com) to gain further insight into the biology of studied traits and their relationships. Although the methodology was applied to beef cattle traits, using high-density SNP chip genotypes, the general framework can be applied to any species, any trait, and it can easily accommodate sequence-level data.

Significant regions emerging from CS were compared with those identified by SUPERGNOVA and LAVA. The CS identified more significant regions affecting the correlations between traits than existing methods, such as SUPERGNOVA and LAVA. This is because the CS does not use local heritability estimates, as found in SUPERGNOVA, or estimate negative local variance, as found in LAVA. The absence of these limitations resulted in more significant regions emerging from the CS approach for the same dataset. Despite differences between our method and SUPERGNOVA or LAVA, six significant local genetic correlations were shared between the three methods, all in the BB population. These regions were all located on chromosome 14. Chromosome 14 regions have been extensively documented as affecting many economically important traits cattle, including fertility traits [37–41]. Chromosome 14 aside, the three methods point to different regions that underpin the correlations between the studied fertility traits. The limited number of overlaps identified between CS and existing methods might be expected as SUPERGNOVA and LAVA are not always in agreement either. In one study, out of the 80 significant loci identified using LAVA, only 6 loci were also identified by SUPERGNOVA [42]. Gerring et al. [42] discussed that the poor overlap between SUPERGNOVA and LAVA might be due to the way the two methods de-correlate association statistics within each semi-independent LD block, which in turn affects the distribution of local genetic association across a particular locus. Therefore, comparing the significant loci across these methods might not provide a robust list of pleiotropic loci for the trait pair investigated.

To compare the three methods, we also estimated the correlation between CS results and results from SUPERGNOVA and LAVA. In a previous study, results from SUPERGNOVA and LAVA had an correlation statistic that ranged between 0.31 and 0.58 [24]. In our datasets, CS seemed to agree with SUPERGNOVA (*r* ranged from 0.46 to 0.77), but it differed from LAVA (*r* ranged from − 0.16 to 0.15). In a typical scenario, correlation statistics stabilize with a sample size of 250 data points [43]. In our comparisons between CS and existing methods, only one comparison had a sample size smaller than 250 data points (i.e., AGECL vs IGF1b in BB cattle). For all other scenarios, the sample size was reasonable. The agreement observed between CS and SUPERGNOVA was higher than between CS and LAVA. This difference could be due to the model assumptions and parameter estimations that underpin the three methods. While CS and SUPERGNOVA are similar in model assumptions, they differ from LAVA. Both CS and SUPERGNOVA assume that the vector of joint genetic effects for a given phenotype is random, whereas LAVA assume a fixed effect model. Werme et al. [24] showed that there may be substantial differences between the estimate of local correlation from LAVA and SUPERGNOVA. Our approach of generating SNP effects follows the same model assumption (i.e., random effect model) as SUPERGNOVA and could be the reason for the higher agreement observed (i.e., higher correlation).

The CS approach may facilitate research collaboration, and the re-use of GWAS data. Access to individual-level genotype and phenotype data continues to be difficult due to consent and privacy concerns, and data ownership restrictions [43–45]. For these reasons, GWAS summary statistics are used to estimate global and local correlations in human genetics [22, 24, 25, 44]. GWAS summary statistics are widely available in public repositories, thus allowing data to be shared among researchers for novel discoveries on the genetic basis of complex traits. In animal genetics, contractual obligations, the commercial value of the data, and intellectual property issues hinders genotype and phenotype data to be shared among researchers or public databases. It is more practical to use published SNP effects that emerged from genomic BLUP analyses. SNP effects have been used to estimate global genetic correlations before [46] and now we have expanded that concept to create the CS and estimate local correlations. The use of published SNP effects alongside all GWAS summary statistics and the CS approach could pave the way for novel discoveries in animal and human genetics.

Our results agreed with the established notion that multiple loci regulate reproductive traits [47–49]. Also, the mode of action of these loci and the magnitude of their effect varies across the genome. While some regions had no effect on the genetic correlations under investigation, other loci drive or antagonize the relationships between male and female fertility. The identification of driver and antagonizing loci creates opportunities to further understand complexities, synergism, or trade-offs between quantitative traits. For example, correlations estimated from SNP effects have allowed researchers to construct gene networks [46]. Thereby, these types of approaches could contribute to linking genotype with phenotype.

The two beef cattle populations investigated in this study are distinct in terms of their genetic composition. Brahman (BB) cattle are typically of *Bos indicus* origin whereas TC beef cattle emanated from the crossing between *Bos indicus* and *Bos taurus* breed [50]. Despite these differences, we found that a considerable number of annotated genes and QTLs driving trait correlation overlaps across breeds, although with variations in the size of SNP effects. This corroborates the findings of Bolormaa et al. [51], where a substantial number of QTLs were found segregating in *Bos indicus* and composite cattle using the same dataset. In this present study, the top and most significant genomic signal driving trait correlation across all pairwise traits in BB were located on BTA14. The significant region contains a widely known and well-characterized QTLs, including the *PLAG1* gene*,* reported to be associated with growth and reproductive traits in our populations and other studies [37, 38, 39, 40, 41, 52]. In TC however, the top signal differs across traits and mostly spread across two or three chromosomes, although with considerable number of overlaps with BB. This could be partly due to the variations in the architecture of composite breed [53]. The genome of composite breeds usually contains new haplotypes emerging from generations of crossbreeding. Moreover, the contribution of the founder populations on chromosomes and specific genomic regions are usually unevenly distributed, which most likely shapes the genome of composite breeds [53]. In short, differences between BB and TC are likely to impact the results of our analyses. Breed differences are expected, and so when two breeds share a similar result, it enhances our confidence in calling significant windows for the interplay between male and female fertility traits.

Most genomic regions antagonizing the genetic correlations between male and female fertility traits were located on chromosome X. Gene expression on chromosome X differs across-sex, resulting in genomic sexual conflict [54, 55, 56]. Genes in these antagonizing regions include *PO1FB, ZNF711, APOOL, HDX, DACH2, FAM133A,* among others. These genes are associated with different disorders including infertility, reproductive deficiencies, primary ovarian failure [57, 58, 59]. When some of these genes are over-expressed, it can dysregulate the cristae morphology of the mammalian mitochondria [60]. Understanding how these antagonizing genes interact to influence (in)fertility could help improve the reproductive potentials of beef cattle.

In animal production, more research is carried out on milk production-related traits, thereby creating large proportion of records for these traits in the cattle QTL database. These volumes of records can create a bias in the QTLs representativeness [31]. The QTL enrichment analysis allows testing the significance of the QTL representative using chromosome-wide approach to detect specific genomic region with many QTLs for a specific trait. For example, the driver regions for AGECL vs IGF1b in chromosome 14 (BB cattle) co-locate with 33 different QTLs for economically important traits. The recurrent association of BTA14 with multiple traits could suggest complex genetic mechanisms such as pleiotropy, epistasis, hitchhiking effects, linkage disequilibrium etc., regulating these chromosomal regions [61, 62]. Understanding these complex mechanisms within BTA14 could inform how these important regions are used in genomic selection for fertility traits, and hopefully avoiding antagonistic effects on other traits.

Another interesting result from this study is the shared biology between the two breeds relative to the traits under study. Despite breed differences, the enriched reproductive QTLs driving the genetic correlations between male and female fertility are the same for the two cattle populations. Most of the enriched QTLs are related to reproductive traits measured early life. A possible explanation could be that the reproductive phenotypes shared common fundamental biology in the two populations. For the antagonizing regions, however, most of the reproductive QTLs were breed specific depending on the trait pair. Perhaps, this could be partly explained by the diverse genetic composition of the two breeds. Understanding the genomic architectures driving these early-in-life male and female fertility traits and their known genomic antagonisms could foster effective selection for both traits in tropical breeds [63, 64].

Leveraging the directionality of gene effects from our method with IPA knowledge base, several biological pathways known to be involved in reproduction (i.e., studied trait) were significantly enriched for all pairwise traits investigated across the two breeds. These pathways include sperm motility, estrogen receptor signaling, p38 MAPK signaling, GnRH signaling, cAMP-mediated signaling, AMPK signaling, and androgen signaling, among others. Although IPA provided information about the activation or inhibition state for the enriched canonical metabolic pathways with the use of the **r** estimates in place of the gene expression values, we are not sure if these pathways were being activated or inhibited since we don’t have information about the expression values of the genes in these pathways. For example, Rho GDP Dissociation Inhibitor (RHOGDI) pathway was the only significant signaling pathway found to be inhibited across breeds in all pairwise traits investigated using IPA comparison analysis. Numerous studies have also reported that the RHOGDIs protein are involved in sperm movement, sperm capacitation and acrosome reaction, a process that is critical to occur for the sperm to interact and penetrate the egg for fertilization to take place [65, 66, 67]. The knockout of any of the RHODGI genes could results in impaired spermatogenesis in male, implantation problem in females and more severe phenotypes with additional immunological defects [68, 69, 70]. Notably, low reproduction performance is one of the major challenges facing beef producers in Northern Australia [71, 72]. Reproductive wastage is usually common, which is often a result of pregnancy failure and calf mortality [73, 74]. Given the role of the RHODGI pathway in reproduction, future studies could use gene expression data to investigate the genes involved in these pathways as a candidate region for infertility in cattle since we only use the **r** estimates in this study.

Finally, the CS methodology is not without limitations. CS can only analyse local correlations between two phenotypes, unlike LAVA that offers the opportunity to identify local genetic correlations across several traits. As always, using a larger sample size could result in better estimates of SNP effects, which might impact in the CS windows significance. As it stands, the CS uses GBLUP solutions of SNP effects to account for genotype co-variance. There are other methods that could be useful in this framework, such as the Bayesian alphabet [49, 75]. Bayesian methods such as BayesR provide precise estimates of SNP effects that might shrunk less than those from genomic BLUP analyses and might fit the genetic architecture of some traits better [76, 77, 78]. We aim to expand the CS framework to multiple traits and to Bayesian approaches in our future work. This goal is beyond the scope of the current study.

## Conclusion

Overall, the framework developed in this study extends our knowledge about the regions driving and antagonizing correlations between quantitative traits. These regions confirmed the polygenic nature of the traits being studied and pointed to genes of interest. Most of these genes co-localized with known QTLs related to milk production and fertility traits, especially puberty. While the enriched reproductive QTLs driving the genetic correlations between male and female fertility were found to be same for both cattle populations studied, the antagonizing regions were population specific and were mostly mapped to chromosome X. These suggest regions of the chromosome X for further investigation into the trade-offs between male and female fertility. Although the methodology was applied to cattle phenotypes, using high-density SNP genotypes, the general framework developed can be applied to any species or traits, and it can easily accommodate genome sequence data.

## Materials and methods

### Traits, genotypes and estimated genetic correlations

The traits used to demonstrate this methodology are a subset of traits from our previous study [29], where bivariate genetic correlations were estimated between 7 male and 6 female early-in-life reproductive phenotypes in two independent tropical beef cattle populations (BB and TC). The two female traits selected for this study are age at detection of the first *corpus luteum* (AGECL, days) and cows’ blood concentration of insulin growth-factor 1, measured at 18 months of age (IGF1c). Only one male trait was selected: the blood concentration of insulin growth-factor 1, measured at 6 months of age (IGF1b). The selected traits consisted of ~1000 individuals measured precisely through frequent ovarian scanning, i.e., AGECL and laboratory assay i.e., IGF1b and IGF1c. These traits are important in beef cattle fertility, especially during pubertal development. The estimated global genomic correlations between the traits listed above in each population using high-density SNPs have been reported in our previous study [29]. The estimated genomic correlations and their corresponding standard error (S.E), heritability of each trait, number of SNPs and number of animals in each population are provided in Additional file 6 (Table S20). The traits were selected because they had significant estimates of genomic correlation (i.e., traits with standard error (S.E) less than half of the size of the correlation) and different strength or direction of genetic relationships (i.e., negatively, and positively correlated traits).

### Overview of methods

For each trait considered in the two beef cattle populations, we estimated the genomic breeding values (GEBVs) of individuals using the genomic best linear unbiased prediction GBLUP model implemented in GCTA [79]:

1 $$\mathbf{y}=\mathbf{Xb}+\mathbf{Za}+\mathbf{e}$$

where *y* is the vector of phenotypes, X is the incidence matrix of fixed effects, *b* is the vector of fixed effects, Z is the design matrix assigned to GEBV*, a* is the vector of GEBVs for each animal, and *e* is the vector of residuals. Vectors a and e are assumed to follow a normal distribution, thus a ∼ N (0, Gσ_g_^2^⁠) and e ∼ N (0, Iσ_e_^2^)⁠. The general solution of the mixed model equation is in the form:

2 $$\left(\begin{array}{cc}\mathbf{X}\hbox{'}{\mathbf{R}}^{-\mathbf{1}}\mathbf{X}& \mathbf{X}\hbox{'}{\mathbf{R}}^{-\mathbf{1}}\mathbf{Z}\\ {}\mathbf{Z}\hbox{'}{\mathbf{R}}^{-\mathbf{1}}\mathbf{X}& \mathbf{Z}\hbox{'}{\mathbf{R}}^{-\mathbf{1}}\mathbf{Z}+{\mathbf{G}}^{-\mathbf{1}}\boldsymbol{\upalpha} \end{array}\right)\left(\begin{array}{l}\mathbf{b}\\ {}\mathbf{a}\end{array}\right)=\left(\begin{array}{l}\mathbf{X}\hbox{'}{\mathbf{R}}^{-\mathbf{1}}\mathbf{Y}\\ {}\mathbf{Z}\hbox{'}{\mathbf{R}}^{-\mathbf{1}}\mathbf{Y}\end{array}\right)$$

Here, G^− 1^ is the inverse matrix of the variance-covariance matrix of random effects, which indicates the SNP-SNP variance-covariance matrix i.e., the GRM and was constructed following Method 1 of VanRanden [80]. α is the shrinkage factor, calculated as ratio of the residual variance (σ_e_^2^) to the additive variance (σ_g_^2^). The full details of the fixed effects used in the model have been reported in our previous study [29]. For AGECL, the contemporary groups (i.e., cohort of animals born in the same year and raised together under the same management conditions) were used as the fixed effect. For IGF1c and IGF1b, the contemporary groups and the age of animals at the time if measurement were used as fixed effect. We used the first two principal components in addition to the GRM to account for the, quite varied, breed composition in the TC breed, for these traits.

Following the estimation of GEBVs for each animal in each trait, we then back-solved these GEBVs to obtain SNP effects for all chromosomes following the method illustrated by Strandén and Garrick [81];

$$\hat{\boldsymbol{u}}=\frac{\mathbf{1}}{\sum_{\boldsymbol{j}=\mathbf{1}}^{\boldsymbol{m}}\mathbf{2}{\boldsymbol{p}}_{\boldsymbol{j}}\left(\mathbf{1}-\boldsymbol{p}\right)}{\boldsymbol{X}}^{\prime }{\boldsymbol{G}}^{-\mathbf{1}}\hat{\boldsymbol{a}}$$

Where $\hat{\boldsymbol{u}}$ is the vector of estimated SNP effects; *m* is the number of SNPs; ***p***_***j***_ is the allele frequency of the second allele of the jth marker; X is a matrix with gene contents for all markers; G is a genomic relationship matrix and $\hat{\boldsymbol{a}}$ is a vector of GEBVs.

The estimation of GEBVs used a GBLUP method, a mathematical transformation of SNP BLUP [82], which explicitly fits the unscaled correlation matrix among the SNPs [80, 83, 84], in other words, the LD matrix among the SNPs. The SNP effects were then obtained by back-solving as per eq. (3). The solution of SNP effects with this approach intrinsically accounts for LD among SNPs [82, 85, 86] and the effect of a QTL is likely distributed across all SNPs that have a non-random association with the QTL [87, 88]. As a result, the SNP effects are not biased by LD and resulting effect sizes can be considered independent. These SNP effects has been used in GWAS to get insight into the genetic architecture of traits [89, 90] and to estimate direct genomic breeding values (DGV) based on small genomic regions, termed ‘local DGV’ for quantitative trait loci mapping [91, 92].

Using a chromosome-wide approach, we divided the SNP effects on the same chromosome into small sliding windows of 500 SNPs each and then estimated the local correlation (**r**) between traits as being the correlation between the 500-SNP effects estimated for trait A and the 500-SNP effects estimated for trait B. We then moved 100 SNPs further from the start of the previous window to select the next 500-SNP window, which partially overlapped with previous window, hence producing sliding windows that were 100 SNPs distant from the previous window. This was repeated for each trait pair, and for each chromosome, in a chromosome-by-chromosome approach.

For mathematical illustration, the **r** between the first trait (x) and second trait (y) of length N is defined as follows:

$$r=\frac{\sum \limits_{i=1}^N\left({x}_i-\overline{x}\right)\left({y}_i-\overline{x}\right)}{\sqrt{\left[\sum \limits_{i=1}^N{\left({x}_i-\overline{x}\right)}^2\right]\left[\sum \limits_{i=1}^N{\left({y}_i-\overline{y}\right)}^2\right]}}$$

Where x_i_ and y_i_ denote the ith SNP effects of x and y across all chromosomes. Thus, each 500 SNP effects of the 100 sliding window **r** estimates between x and y can be formalized as below;

$$r\left[j\right]=\frac{\sum \limits_{i=j}^{j^i}\left({x}_i-\overline{x}\right)\left({y}_i-\overline{x}\right)}{\sqrt{\left[\sum \limits_{i=j}^{j^i}{\left({x}_i-\overline{x}\right)}^2\right]\left[\sum \limits_{i=j}^{j^i}{\left({y}_i-\overline{y}\right)}^2\right]}}$$

**Fig. 6:**
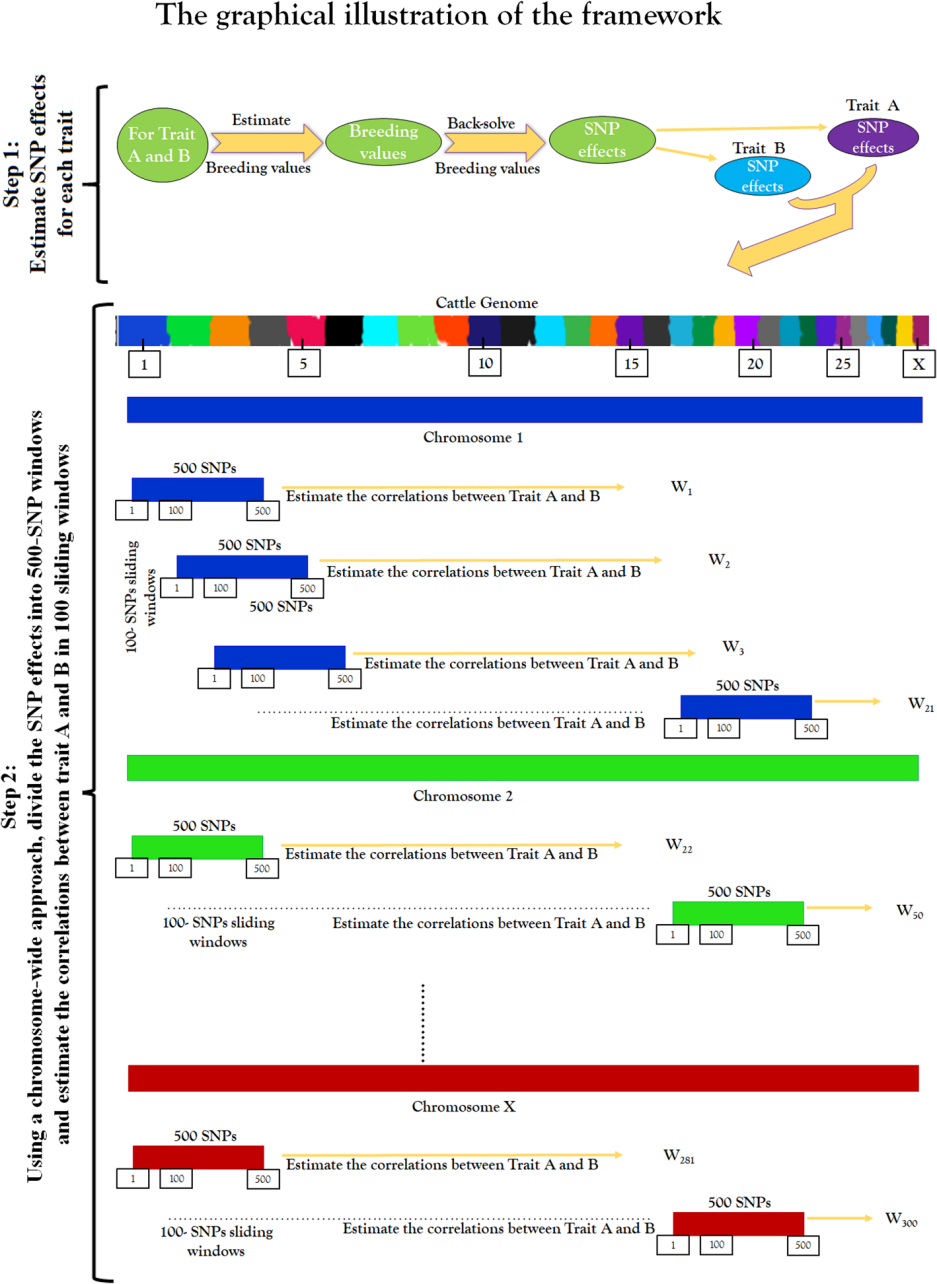
The graphical illustration of the sliding window framework. The framework involves 2 steps. Step 1 start from the estimation of genomic breeding values to the obtainment of SNP effects for each pairwise trait. Step 2 start from the estimation of 500-SNP effects in a chromosome-wide approach to the obtainment of the correlation estimate in a 100-sliding window

Where j ∈ [0, *N – K* + 1] is the start point of each window, K is the window length and j^i^ = j + K-1. The window is then shifted 100 SNP effects away from the start of the previous window and a new **r** is computed for each shift yielding a new estimate for each chromosome. The resulting **r** estimates for all the chromosomes combined were denoted as W_1_….W_n_. The graphical illustration of this framework is presented in Fig. 6.

Depending on the global correlation observed between the traits considered, the driver and antagonizing windows can be deduced. In this study, AGECL and IGF1b were negatively correlated. Hence, the driver windows were windows with significant and negative **r** estimates, while the antagonizings were windows with significant positive **r** estimates. For the positively correlated relationship between IGF1c and IGF1b, the driver windows were windows with significant and positive **r** estimates and the antagonizings were windows with significant and negative **r** estimates.

To showcase that the SNP effects from the back-solve approach are not biased by LD, we pruned the genotypes data of each breed based on pairwise LD to obtain a pruned subset of SNPs that are in approximate linkage equilibrium (LE) using PLINK v.19 [93]. The LD based SNP pruning method was applied with a window size of 500 SNPs, shifting the window by 100 SNPs at the end of each step and removing SNPs with LD > 0.5. The resulting pruned genotypes were then used to re-run the CS methodology as described above.

### Identification of significant windows

Significant window for each **r** estimate were obtain using Fisher’s Z method [94]. Firstly, we performed permutation test by randomly reshuffling the SNP effects in each window across all the chromosomes in 10,000 iterations (p_1_…p_10000_) for each trait. Subsequently, we estimated correlations for 100-sliding windows of 500-SNP effects for each iteration in each window between the two traits as described above. We then estimated the mean of the permutation distribution of the **r** estimates across all the iterations to obtain a single value (r_p_) for each window. Next, the significance between the real estimate r and r_p_ for each window was determined by Fisher’s Z-statistic: for r and r_p_${Z}_r=\frac{1}{2}\mathit{\ln}\left(\frac{1+r}{1-r}\right)$ and ${Z}_{r_p}=\frac{1}{2}\mathit{\ln}\left(\frac{1+{r}_p}{1-{r}_p}\right)$, respectively. This allowed for normal distribution and facilitated the calculation of standard error-based Z-difference between correlation coefficient of both r and r_p_ i.e., *Z*_*r*_*-*_*rp:*_

$${Z}_{r-{r}_p}=\frac{\mid {Z}_r-{Z}_{r_p}\mid }{\sqrt{\frac{1}{n+3}+\frac{1}{n-3}}}$$

Where n is the sample size of the SNP effects for each window, in our case i.e., 500. To retrieve the corresponding *P*-values for each window, we estimated the cumulative distribution function for *Z*_*r*_*-*_*rp*_ while accounting for a two-tail test. The significance level was then set at Bonferroni-corrected *P*-value < 0.05. As a result, significant windows were selected for the drivers and antagonizings genomic regions for each trait pair. Windows that were not significant were tagged “neutral windows” i.e., windows with no effect on the trait pairs. Finally, the **r** estimates of the significant windows for the driver and antagonizing regions were ranked from top to bottom in percentage (%). The rank values were used solely for the purpose of subsequent downstream analyses, i.e., pathway analyses. The ranking was done separately for the driver and antagonizing windows for each pairwise trait investigated in each population.

#### Performance comparison with other methods

To showcase the performance of CS, we compared the results from CS for each trait pair in the two populations with the results SUPERGNOVA and LAVA. Both SUPERGNOVA and LAVA are tools used to estimate local genetic correlation between complex traits in human using GWAS summary statistics.

For illustration, consider two traits Y_1_ and Y_2_ with sample size n_1_ and n_2_, respectively. The standardized trait values of Y_1_ and Y_2_ follows the linear model below: ${Y}_1=\sum_{i=1}^i{\boldsymbol{X}}_i{\boldsymbol{\beta}}_i+\boldsymbol{\epsilon}$ and ${Y}_2=\sum_{i=1}^i{\boldsymbol{Z}}_i{\boldsymbol{\gamma}}_i+\boldsymbol{\delta}$. The ***X***_*i*_ and ***Z***_*i*_ are standardised genotype matrices and ***β***_*i*_ and ***γ***_*i*_ are the vector of joint genetic effects in region *i*. ϵ and δ are the error terms.

SUPERGNOVA assumes the vector of joint genetic effect for the traits to be random and follows a multivariate normal distribution. The genetic covariance (*p*) between the two traits in region *i* is then estimated by minimizing the distance between the empirical covariance of Z scores [25]. LD matrix for region *i* are estimated from an external reference panel (e.g., the 1000 Genomes Project using LDdetect [25, 95]. To account for sample overlap, the first K_i_ eigenvectors, which is determined adaptively in SUPERGNOVA, are used to transform and decorrelate Z scores in any given region *i*. Subsequently, local genetic covariance in each region is estimated using a weighted least squares regression [25]. Finally, local genetic correlation in a region *i* is estimated by $\frac{p_i}{\sqrt{h_{1_i}^2}{h}_{2_i}^2}$, where *p*_*i*_ is the local genetic covariance and ${h}_{1_i}^2$ and ${h}_{2_i}^2$ are the heritability estimates of trait 1 and 2 at the region *i.*

In contrast to SUPERGNOVA, LAVA assumes the vector of joint genetic effects ***β***_***i***_ and ***γ***_*i*_ for the two traits to be fixed rather than random [24]. To estimate the *p*_*i*_, LAVA apply Method of Moments approach [96] using the computed standardize principal components and the matrix of the genetic effects for a given region *i*. Thus, LAVA differs from SUPERGNOVA in both the underlying model assumption and the parameter estimation [24]. Finally, local genetic correlation in a region *i* is estimated as the ratio of scaled covariance of the genetic components for trait 1 and 2 to the square root of the scaled variance of the genetic component of trait 1 and trait 2 in that region.

For these comparisons, we performed a single-trait GWAS for each of the trait to obtain the summary statistics data to be used for SUPERGNOVA and LAVA. Instead of using independent LD blocks as described in the two methods, we partitioned each chromosome to align with our sliding window methodology of 500 SNP effects with 100-sliding windows for equal comparison. Where applicable, we used the genotype data se external reference panel. To ensure that all loci in LAVA were analysed irrespective of whether they are signalled or not, we set the univariate threshold to 1 and use the default param.limit of 1.25.

#### Gene and Quantitative traits loci (QTL) annotation

The significant windows along with their corresponding chromosome coordinates, **r** estimates and rank values for the driver and antagonizing regions that passed the specified threshold criteria following the Fisher’s Z method in BB and TC were selected. The selected windows were used for gene and QTL annotation using R package GALLO: Genomic Annotation in Livestock for positional candidate Loci (https://CRAN.R-project.org/package=GALLO) [31]. The .gtf annotation file corresponding to the bovine gene annotation from ARS-UCD1.2 assembly and the .gff file with the QTL information from cattle QTL Database (https://www.animalgenome.org/cgi-bin/QTLdb/index [97, 98]), were used for gene and QTL annotation, respectively [31]. The two files use the same bovine reference genome (ARS-UCD1.2) to map the gene and QTLs. A remarkable advantage of GALLO is that the software retains all the information present in the input file when producing the output file. As a result, genes within each window can retain their **r** estimates and the rank values specific for their window.

The number and percentage of genes and QTLs annotated within a population (BB or TC) and the overlaps across populations (BB and TC) were investigated. Furthermore, we examined the QTLs representativeness and diversity to explain better the genomic content of the significant windows for the driver and antagonizing regions. Hence, the visualization of the percentage of cattle QTL classes from the cattle QTL database (i.e., milk, reproduction, production, meat and carcass, health and exterior) were plotted using a pie chart by GALLO (27).

### QTL enrichment analysis

To further test the significance of the QTLs, a within population QTL enrichment analysis was conducted using a chromosome-based approach. The QTL enrichment analysis, using all the QTL information annotated within the significant windows for the driver and antagonizing regions, was performed using the qtl_enrich function from GALLO [99, 100]. Briefly, the observed number of QTLs for each trait in each annotated chromosome were compared with the expected number using a hypergeometric test approach in a 1000 iteration rounds of random sampling from the entire cattle QTL database. With this approach, a *P*-value for the QTL enrichment status of each annotated QTLs within the significant windows was estimated. These estimated *P*-values were corrected for multiple testing using a false discovery rate (FDR) of 5%. In addition, we used chord plots to reveal the relationships between the two breeds for the enriched reproductive QTLs based on the driver and antagonizing genomic regions.

### Functional enrichment analysis

The annotated genes along with their corresponding **r** estimates and rank values for the significant driver and antagonizing windows for each pairwise trait in BB and TC populations were subjected to enrichment analysis using the commercial QIAGEN’s Ingenuity Pathway Analysis (IPA; v.8.8, http://www.ingenuity.com). The IPA allows identifying overrepresented biological mechanism, metabolic pathways, and diseases and biological functions that are highly relevant to the traits of interest using the directionality of the submitted gene list [101, 102]. From out outcome of our methodology, genes within each window come with their directionalities, in this case, **r** estimates. Thus, we leveraged on the directionality of each gene by allowing the driver genes to be upregulated and antagonizing genes to be downregulated.

Summarily, a merged dataset containing gene identifiers that were significant for both the driver and antagonizing windows for each pairwise trait in each population and their corresponding **r** estimates and rank values were uploaded into IPA. The **r** estimates were used as the “Expr Log Ratio” and the rank values (see identification of significant windows in methods) were used as *P*-values. The IPA software recognizes gene with positive signs (+) for “Expr Log Ratio” as upregulated genes and negative sign (−) as downregulated genes. We aim to allow the driver gene lists to have positive values for “Expr Log Ratio” and the antagonizing gene lists to be negative. Where this is not achievable based on the original **r** estimates (i.e., AGECL vs IGF1b, where the drivers are negative and the antagonizers are positive), we reversed the sign for the driver and antagonizing genes to meet this objective.

Of note, IPA can only analyse a maximum of 8000 gene list. In most cases, the merged gene list for each trait pair in each population is often > 8000. Hence, we used the rank values as the cut-off to select the top ~ 80% genes from the driver and antagonizing gene list for the pathway analyses whenever the gene list is more than 8000. Using a proportion of the gene list to infer biological pathways might result in the loss of some important biological information relevant to the trait of interest. We analysed the driver and antagonizing gene list separately for each pairwise trait in each population to ensure no important information was lost because of the cut-offs. Further, we compare the result of the separate analyses with the merged gene list from the ~80% cut-off.

The pathway analysis was conducted using the “Core Analysis” function implemented within IPA. In this analysis, associations were calculated using direct and indirect relationships among the gene lists. At first, the gene lists were mapped to human gene data. Genes without an associated gene symbol or gene annotation were subjected to an annotation by homology using BioMart application available in the Ensembl database (http://www.ensembl.org/biomart/martview/) [103, 104]. With this approach, we only considered non-annotated genes with percentage of identity ≥80% with human homolog. Genes with duplicate gene names were removed from the list. The final datasets used for the IPA analyses are presented in Additional file 11 (Table S29-32). Finally, the “Core Analysis” was used to identify canonical metabolic pathways enriched at Benjamini–Hochberg corrected *P*-values (B-H-*P*-value) of *p* < 0.01.

#### Code availability

CS was implemented as a Perl package, which is publicly available at the GitHub repository (https://github.com/optimist0372/Correlation-Scan). Analysis script and the exact package version (v0.05) used to generate the main results can be downloaded from https://github.com/optimist0372/Correlation-Scan.

## Supplementary Information

**Additional file 1.** The number of windows, chromosome number, chromosome coordinates, and correlation estimates of each window for the two trait pairs in Brahman and Tropical Composite population (Table S1-S2).

**Additional file 2.** The genome plot of the LD pruned sliding window correlation estimates for the trait pairs in Brahman (BB) and Tropical Composite (TC) (Fig. S1).

**Additional file 3.** The correlation estimates from SUPERGNOVA and LAVA portioned using 500 SNPs windows with sliding windows of 100 SNPs for all trait pairs in Brahman and Tropical Composite population. (Table S3-S10).

**Additional file 4.** The number of significant driver and antagonizing windows for each trait pair in Brahman and Tropical Composite population (Table S11).

**Additional file 5.** The number of windows, chromosome number, chromosome coordinates, correlation estimates and rank value for the drivers and antagonizing regions (Bonferroni corrected *p* < 0.05) in Brahman (BB) and Tropical Composite population for the studied trait (Table S12-19).

**Additional file 6.** The global genomic correlation estimates, heritabilities (standard error in parenthesis), number of animals and number of SNP from previous study in Brahman and Tropical Composite population (Table S20).

**Additional file 7.** The percentage of QTL type and trait related to reproduction QTLs for the QTL annotation results obtained for the drivers and anatgonizing regions of the two trait pairs in Brahman (BB) and Tropical Composite (TC) population (Fig. S2-S3).

**Additional file 8.** The enriched QTLs of the driver and antagonizing regions for all trait pairs in Brahman and Tropical Composite cattle. The enriched QTLs are rank based on the adj.pval (Table S21-28).

**Additional file 9.** The chord plot showing the relationship between the top 10 enriched reproductive QTLs between Brahman (BB) and Tropical Composite (TC) for the driver (top) and the antagonizing (bottom) regions of the studied traits (Fig. S4).

**Additional file 10.** Canonical pathways significantly enriched (Benjamini-Hochberg *P*-values < 0.01) for all trait pairs in Brahman and Tropical Composite population (Fig. S5-S12).

**Additional file 11.** The final dataset used for Ingenuity Pathway Analysis (IPA) for all trait pairs in Brahman and Tropical Composite cattle (Table S29-32).

## Data Availability

The datasets of the BLUP solution of SNP effects generated and analysed during the current study are available in the Mendeley data repository, 10.17632/hd48y8m3c3.1

## Abbreviations

AGECL: Age at first *corpus luteum*
BB: Brahman
BTA: Bovine chromosome
CS: Correlation Scan
GBLUP: Genomic Best Linear Unbiased Prediction
GEBV: Genomic estimated breeding values
IGF1b: Serum levels of insulin growth hormone measured in bulls at 6-months of age
IGF1c: Serum levels of insulin growth hormone measured in cows at 18-months of age
IPA: Ingenuity Pathway Analysis
LD: Linkage Disequilibrium
QTLs: Quantitative Trait Loci
R: Correlation estimate
SNPs: Single Nucleotide Polymorphisms
TC: Tropical Composite
