# Supplementary material for: Correlation scan: identifying genomic regions that affect genetic correlations applied to fertility traits
Source: BMC Genomics. 2022 Oct 5;23:684. doi: 10.1186/s12864-022-08898-7 (PMC9533527; doi:10.1186/s12864-022-08898-7)
Supplement: Supplementary file 2 — Additional file 2. The genome plot of the LD pruned sliding window correlation estimates for the trait pairs in Brahman (BB) and Tropical Composite (TC) (Fig. S1). [file 12864_2022_8898_MOESM2_ESM.docx]

| 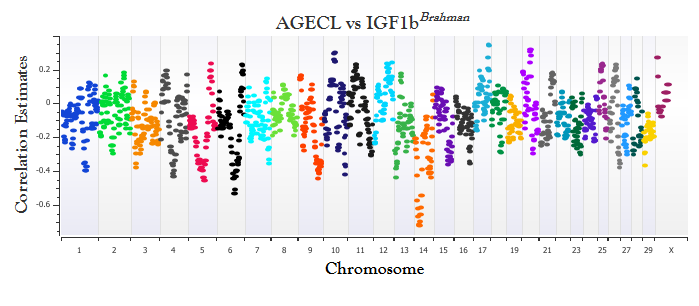  **B** |
| --- |
| 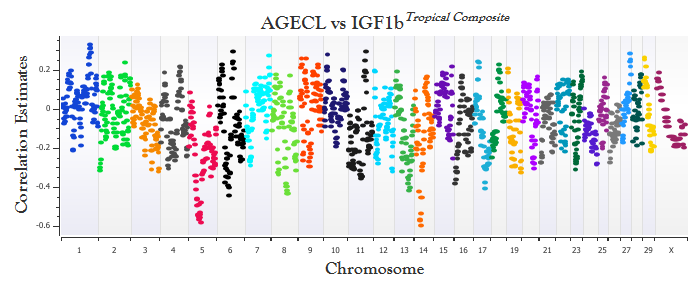 |
| 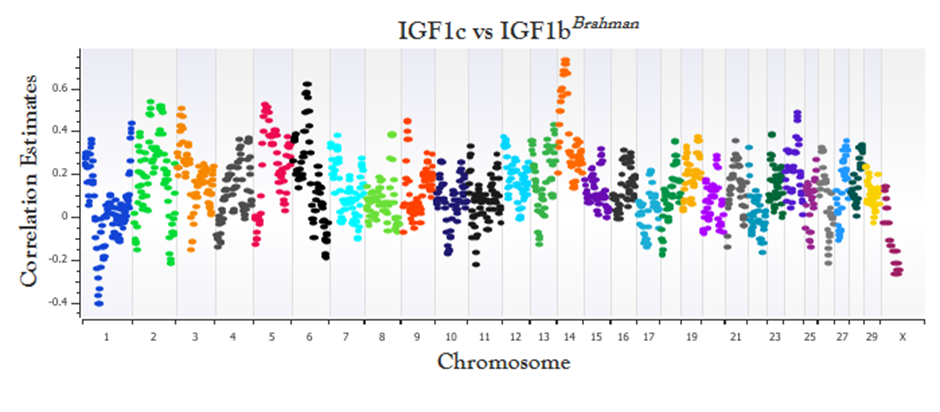  **C** |
| 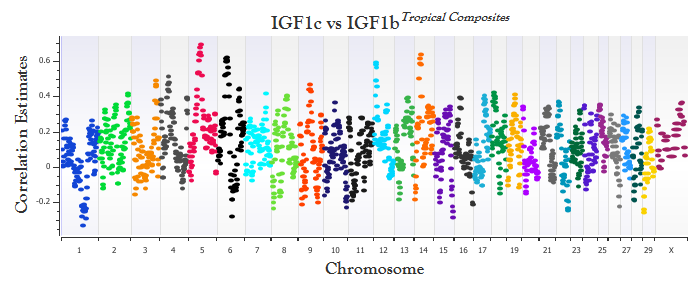  **D** |

**Figure S1.** The genome plot of the LD pruned sliding window correlation estimates for the pairwise traits (BB-AGECL vs IGF1b; **A**, TC-AGECL vs IGF1b; **B**, BB-IGF1c vs IGF1b; **C**, TC- IGF1c vs IGF1b; **D**) in Brahman (BB) and Tropical Composite (TC).  **AGECL**, age at first corpus; **IGF1**, serum levels of insulin growth hormone (measured in bulls, **IGF1b**, or cows, **IGF1c**). The correlation estimates were plotted on the y-axis and the genomic position (i.e., midpoint between the start and end position of each window) of each chromosome on the x-axis, according to the ARS_UCD1.2 bovine reference genome.

**A**
