# Supplementary material for: Correlation scan: identifying genomic regions that affect genetic correlations applied to fertility traits
Source: BMC Genomics. 2022 Oct 5;23:684. doi: 10.1186/s12864-022-08898-7 (PMC9533527; doi:10.1186/s12864-022-08898-7)
Supplement: Supplementary file 4 — Additional file 4. The number of significant driver and antagonizing windows for each trait pair in Brahman and Tropical Composite population (Table S11). [file 12864_2022_8898_MOESM4_ESM.docx]

**Table S11: The number of significant driver and antagonizing windows for each trait pair in Brahman and Tropical Composite population.**

| Pairwise Trait | Number of windows (percentage to the total number) | | Total number significant windows |
| --- | --- | --- | --- |
|  | Driver (%) | Antagonizing (%) |  |
| *Brahman* | | | |
| AGECL vs IGF1b | 1,022 (78.31%) | 283 (21.69%) | 1,305 |
| IGF1c vs IGF1b | 1,293 (85.18%) | 225 (14.82%) | 1,518 |
| *Tropical Composite* | | | |
| AGECL vs IGF1b | 1,230 (71.06%) | 501 (28.94%) | 1,731 |
| IGF1c vs IGF1b | 1,765 (83.93%) | 338 (16.07%) | 2,103 |

**AGECL**, age at first *corpus;* **IGF1c**, serum levels of insulin growth hormone measured in cow; **IGF1b**, serum levels of insulin growth hormone measured in bulls.
