# Supplementary material for: Correlation scan: identifying genomic regions that affect genetic correlations applied to fertility traits
Source: BMC Genomics. 2022 Oct 5;23:684. doi: 10.1186/s12864-022-08898-7 (PMC9533527; doi:10.1186/s12864-022-08898-7)
Supplement: Supplementary file 6 — Additional file 6. The global genomic correlation estimates, heritabilities (standard error in parenthesis), number of animals and number of SNP from previous study in Brahman and Tropical Composite population (Table S20). [file 12864_2022_8898_MOESM6_ESM.docx]

**Table S20:** **The global genomic correlation estimates, heritabilities (standard error in parenthesis), number of animals and number of SNPs from previous study in Brahman and Tropical Composite population**

| Pairwise traits | No of animals | | Estimated Heritability (s.e) | Number of SNP | Genetic  correlation (s.e) |
| --- | --- | --- | --- | --- | --- |
|  | | *Brahman* | | | |
| AGECL vs IGF1b | AGECL- 980  IGF1b- 964 | | 0.56 (0.08)  0.43 (0.07) | 554K | -0.65 (0.13) |
| IGF1c vs IGF1b | IGF1c- 995  IGF1b- 964 | | 0.46 (0.08)  0.43 (0.07) | 554K | 0.86 (0.11) |
|  | | *Tropical Composite* | | | |
| AGECL vs IGF1b | AGECL-996 IGF1b- 998 | | 0.46 (0.08)  0.48 (0.07) | 686K | -0.55 (0.14) |
| IGF1c vs IGF1b | IGF1c- 1015  IGF1b- 998 | | 0.42 (0.09)  0.48 (0.07) | 686K | 0.93 (0.11) |

AGECL, age at first *corpus;* IGF1c, serum levels of insulin growth hormone measured in cow; IGF1b, serum levels of insulin growth hormone measured in bulls; SNP, Single Nucleotide Polymorphisms; s.e, standard error
