# Supplementary material for: Correlation scan: identifying genomic regions that affect genetic correlations applied to fertility traits
Source: BMC Genomics. 2022 Oct 5;23:684. doi: 10.1186/s12864-022-08898-7 (PMC9533527; doi:10.1186/s12864-022-08898-7)
Supplement: Supplementary file 7 — Additional file 7. The percentage of QTL type and trait related to reproduction QTLs for the QTL annotation results obtained for the drivers and anatgonizing regions of the two trait pairs in Brahman (BB) and Tropical Composite (TC) population (Fig. S2-S3). [file 12864_2022_8898_MOESM7_ESM.docx]

| **AGECL-IGF1b-Driver** | |
| --- | --- |
| 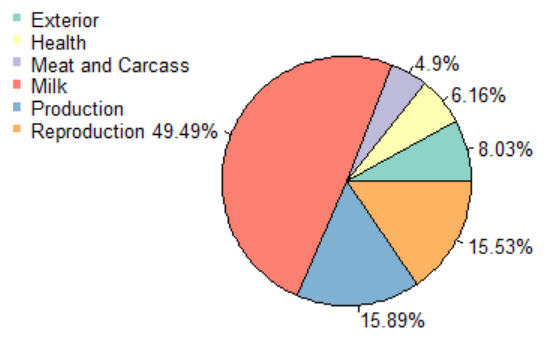  BB | 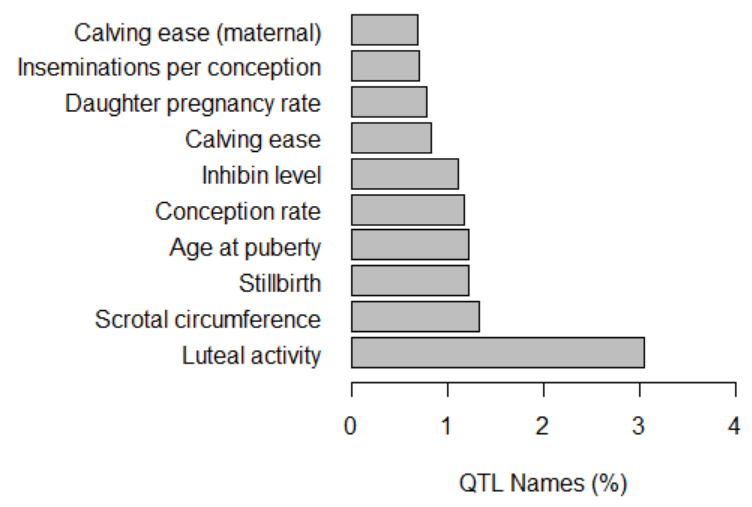 |
| 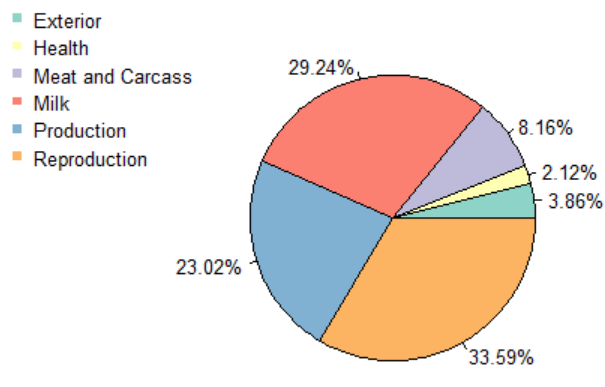  TC | 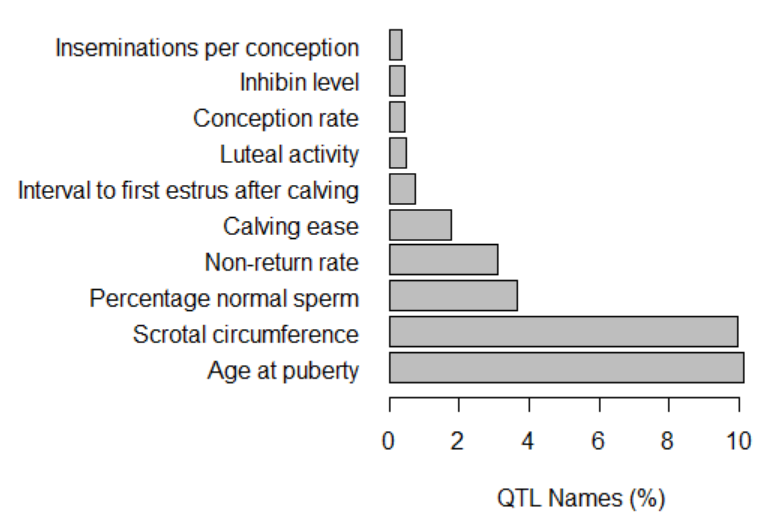 |
| **AGECL-IGF1b-Antagonizing**  **B** | |
| 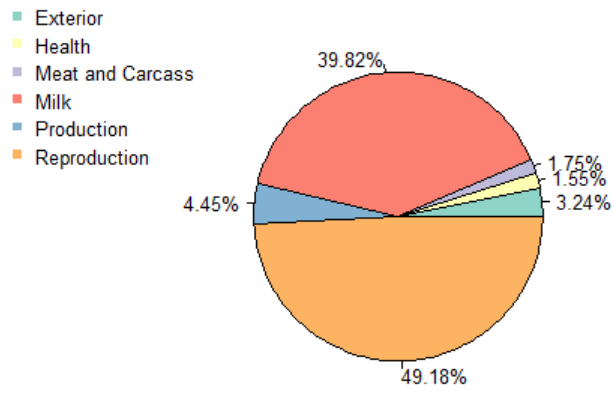  BB | 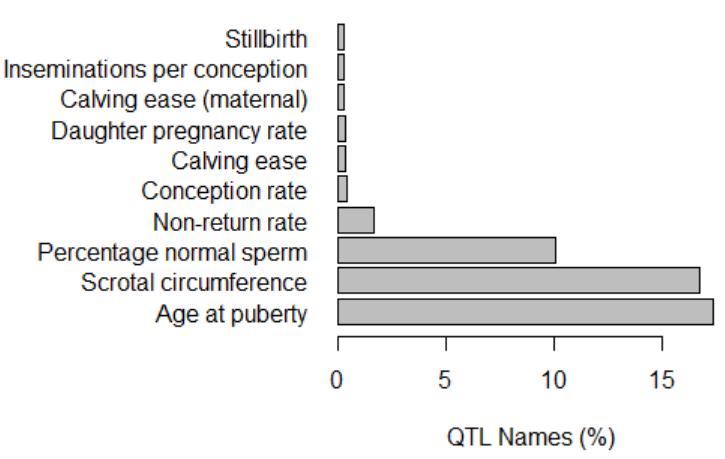 |
| 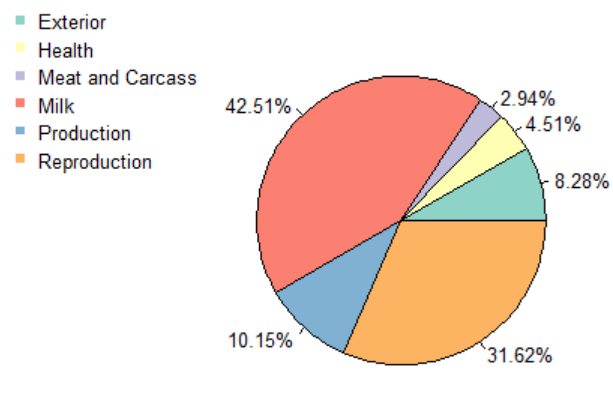  TC | 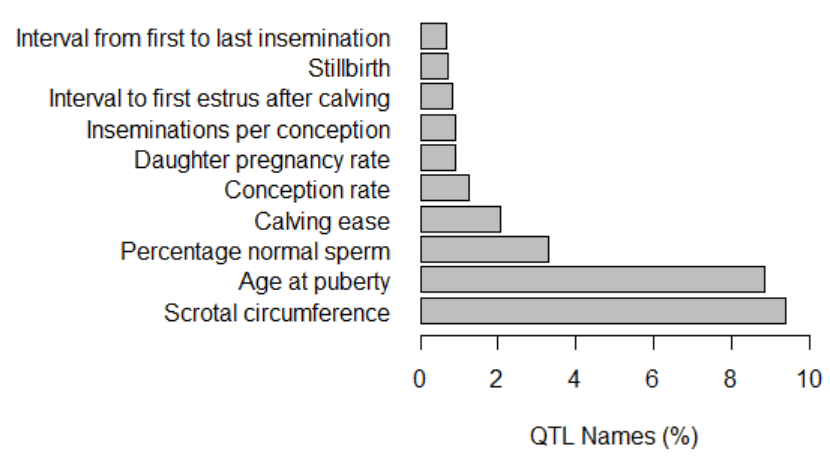 |

**Figure S2.** The percentage of QTL type (pie chart) and trait related to reproduction QTLs (barplots) for the QTL annotation results obtained for (A) AGECL vs IGF1b - driver, (B) AGECL vs IGF1b- antagonizing in Brahman (BB) and Tropical Composite (TC) population. **AGECL**, age at first corpus luteum, **IGF1**, serum levels of insulin growth hormone (measured in bulls, **IGF1b**, or cows, **IGF1c**).

**A**

| **AGECL-IGF1b-Driver** | |
| --- | --- |
| 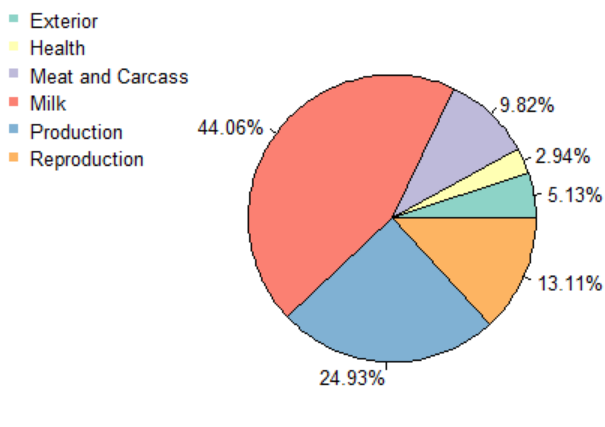  BB | 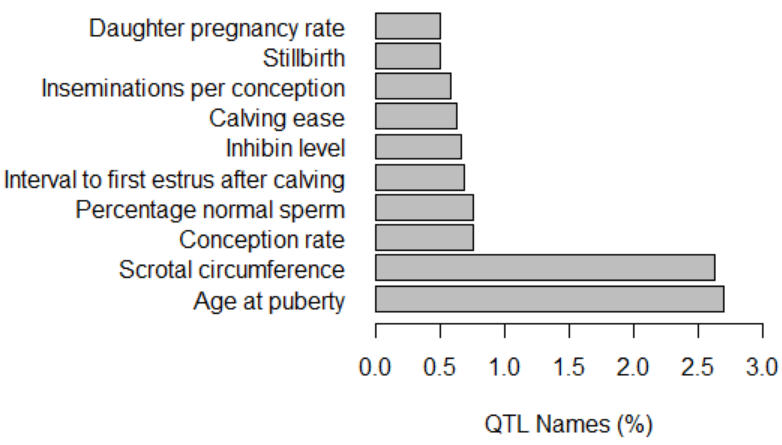  **A** |
| 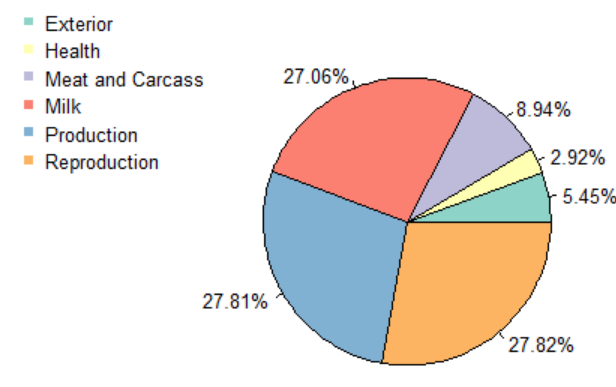  TC | 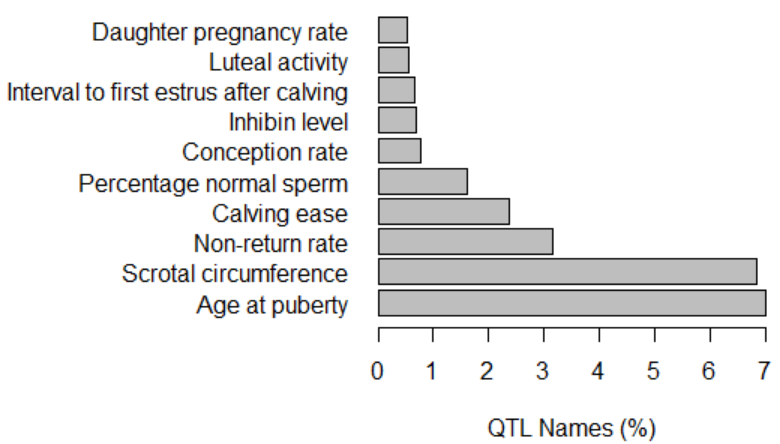 |
| **AGECL-IGF1b-Antagonizing**  **B** | |
| 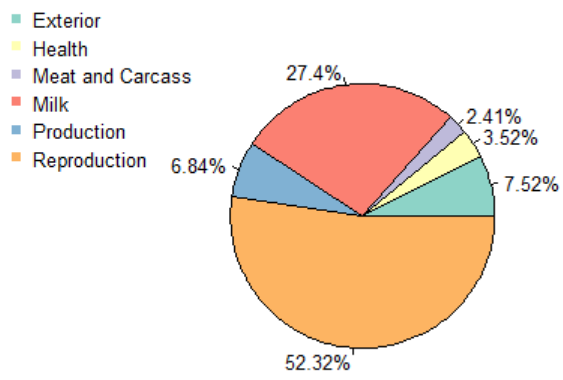  BB | 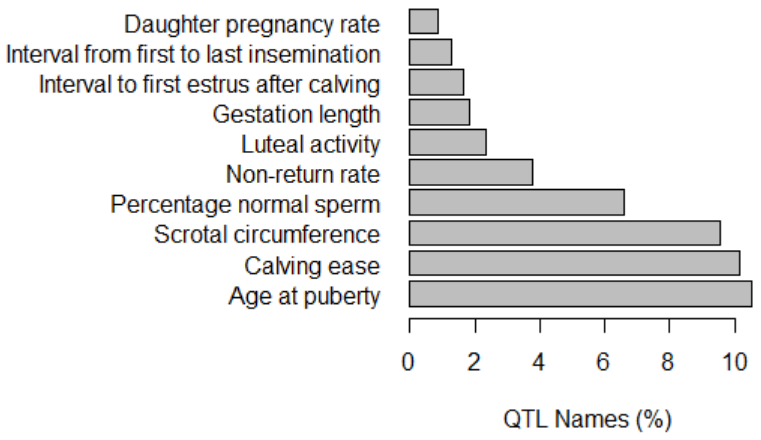 |
| 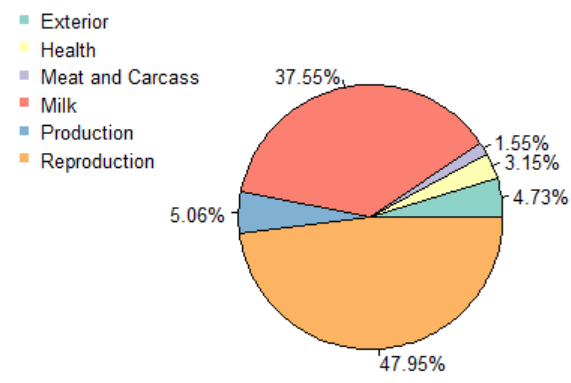  TC | 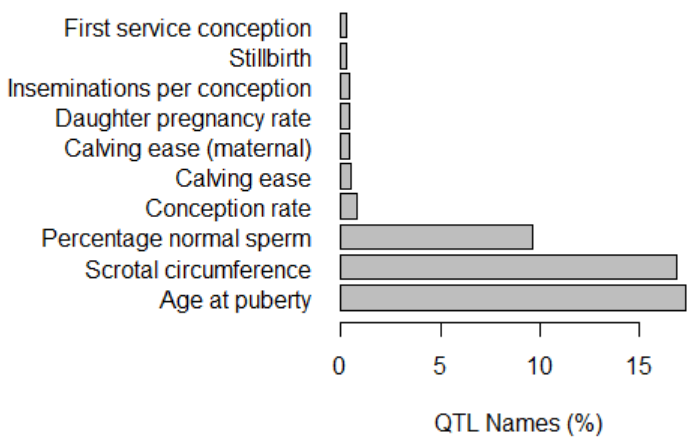 |

**Figure S3**. The percentage of QTL type (pie chart) and trait related to reproduction QTLs (bar plots) for the QTL annotation results obtained for **(A)** IGF1c vs IGF1b- driver, (B) IGF1c vs IGF1b- antagonizing in Brahman (BB) and Tropical Composite (TC) population. **AGECL**, age at first corpus luteum, **IGF1**, serum levels of insulin growth hormone (measured in bulls, **IGF1b**, or cows, **IGF1c**).
