# Supplementary material for: Correlation scan: identifying genomic regions that affect genetic correlations applied to fertility traits
Source: BMC Genomics. 2022 Oct 5;23:684. doi: 10.1186/s12864-022-08898-7 (PMC9533527; doi:10.1186/s12864-022-08898-7)
Supplement: Supplementary file 9 — Additional file 9. The chord plot showing the relationship between the top 10 enriched reproductive QTLs between Brahman (BB) and Tropical Composite (TC) for the driver (top) and the antagonizing (bottom) regions of the studied traits (Fig. S4). [file 12864_2022_8898_MOESM9_ESM.docx]

**Driver**

| 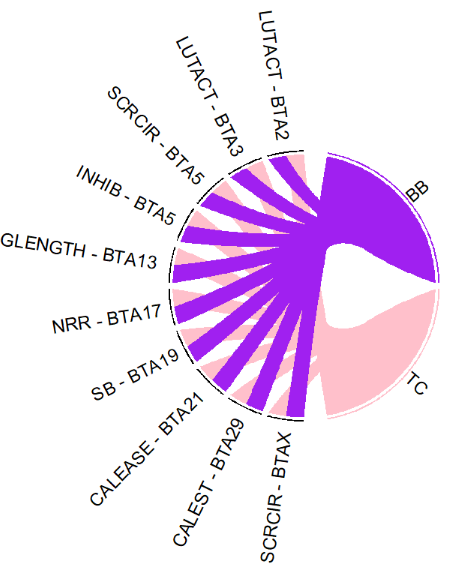 | 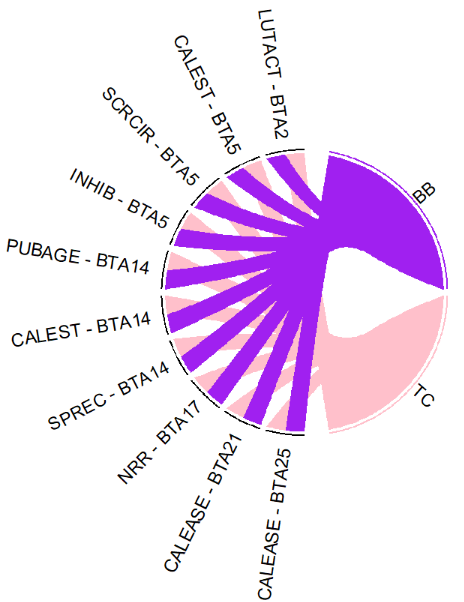  **IGF1c vs IGF1b**  **AGECL vs IGF1b** |  |
| --- | --- | --- |
| 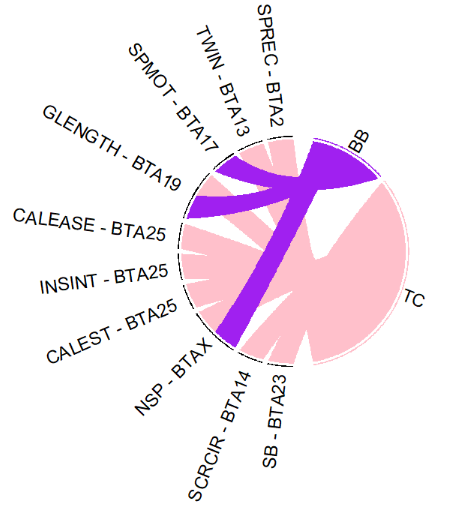  **AGECL vs IGF1b** | 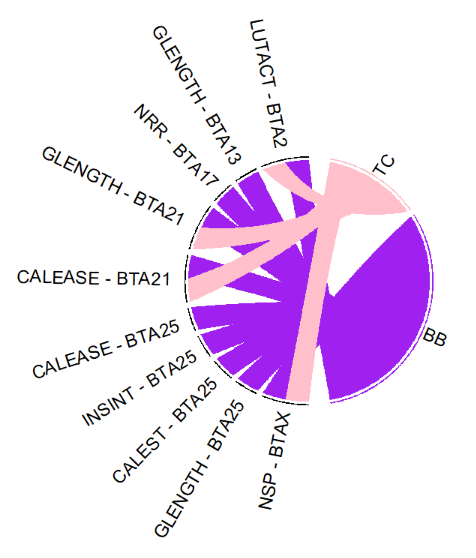  **Antagonizing**  **IGF1c vs IGF1b** |  |

**Figure S4.** The chord plot showing the relationship between the top 10 enriched reproductive QTLs between Brahman (BB) and Tropical Composite (TC) for the driver (top) and the antagonizing (bottom) regions of the studied traits. **AGECL**, age at first corpus luteum, **IGF1c**, serum levels of insulin growth hormone measured in cow; IGF1b. LUTACT, Luteal activity; SCRCIR, Scrotal circumference; INHIB, Inhibin level; PUBAGE, Age at puberty; SPREC, Sexual precocity; NRR, Non-return rate; CALEASE, Calving ease; INSINT, Interval from first to last insemination; SB, Still birth; CALEST, Interval to first estrus after calving; GLENGTH, Gestation length, NSP, Percentage normal sperm.
