# Supplementary material for: Correlation scan: identifying genomic regions that affect genetic correlations applied to fertility traits
Source: BMC Genomics. 2022 Oct 5;23:684. doi: 10.1186/s12864-022-08898-7 (PMC9533527; doi:10.1186/s12864-022-08898-7)
Supplement: Supplementary file 11 — Additional file 11. The final dataset used for Ingenuity Pathway Analysis (IPA) for all trait pairs in Brahman and Tropical Composite cattle (Table S29-32). [file 12864_2022_8898_MOESM11_ESM.pdf]

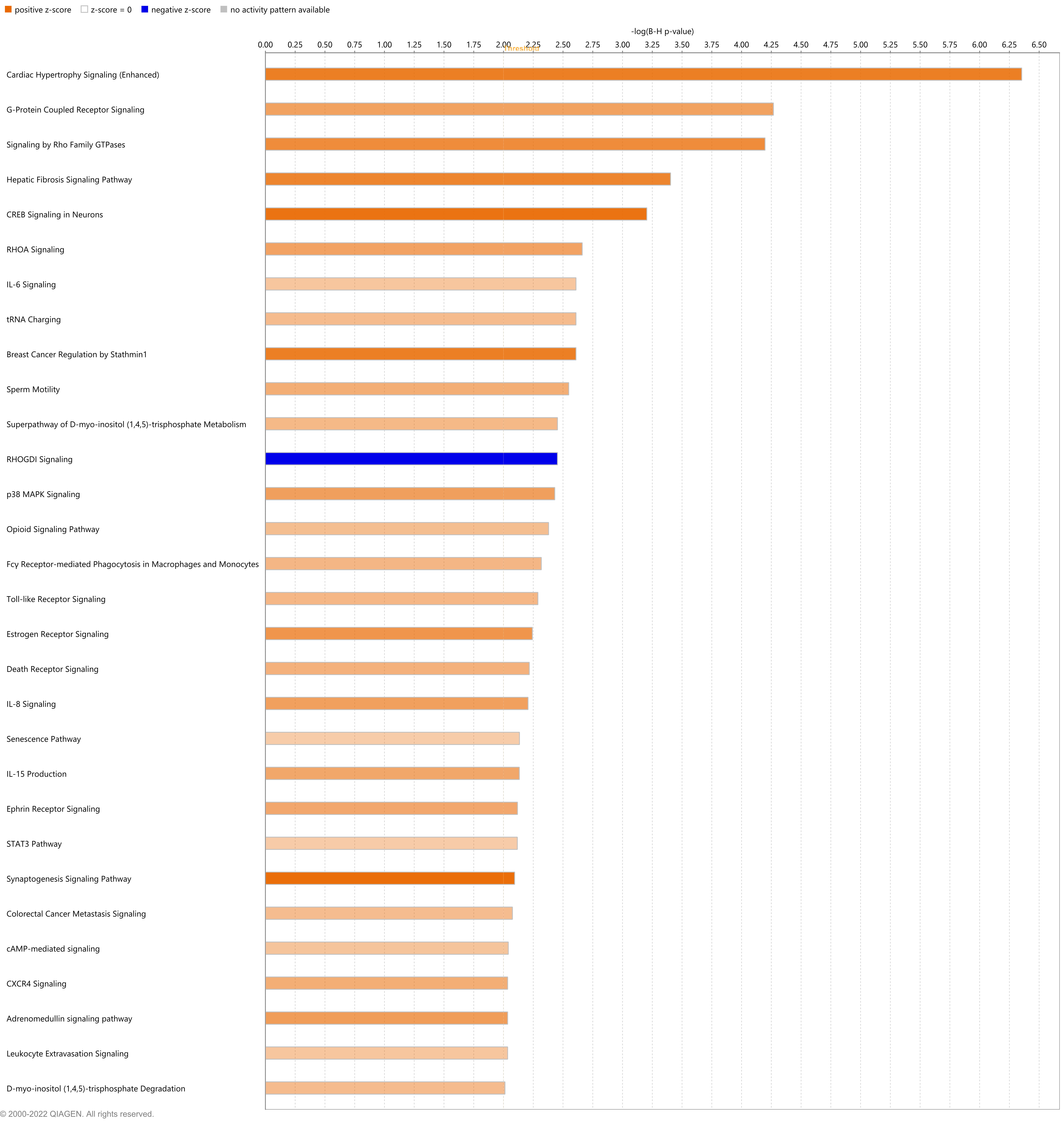

© 2000-2022 QIAGEN. All rights reserved.

Figure S5. Canonical pathways significantly enriched for AGECL vs IGF1b in Brahman population. Significantly enriched canonical pathways were identified using Benjamini-Hochberg pvalues <0.01. AGECL, age at first corpus; IGF1b, serum levels of insulin growth hormone measured in bulls).The left hand side is the enriched canonical pathway and the right hand side is the predicted activation state of the canonical pathway using a z-score. Orange color indicate a positive z-score (Z>2) denoting the activation of the pathway. Blue color indicate a negative z-score (Z<-2) indicating the inhibition of the pathway.

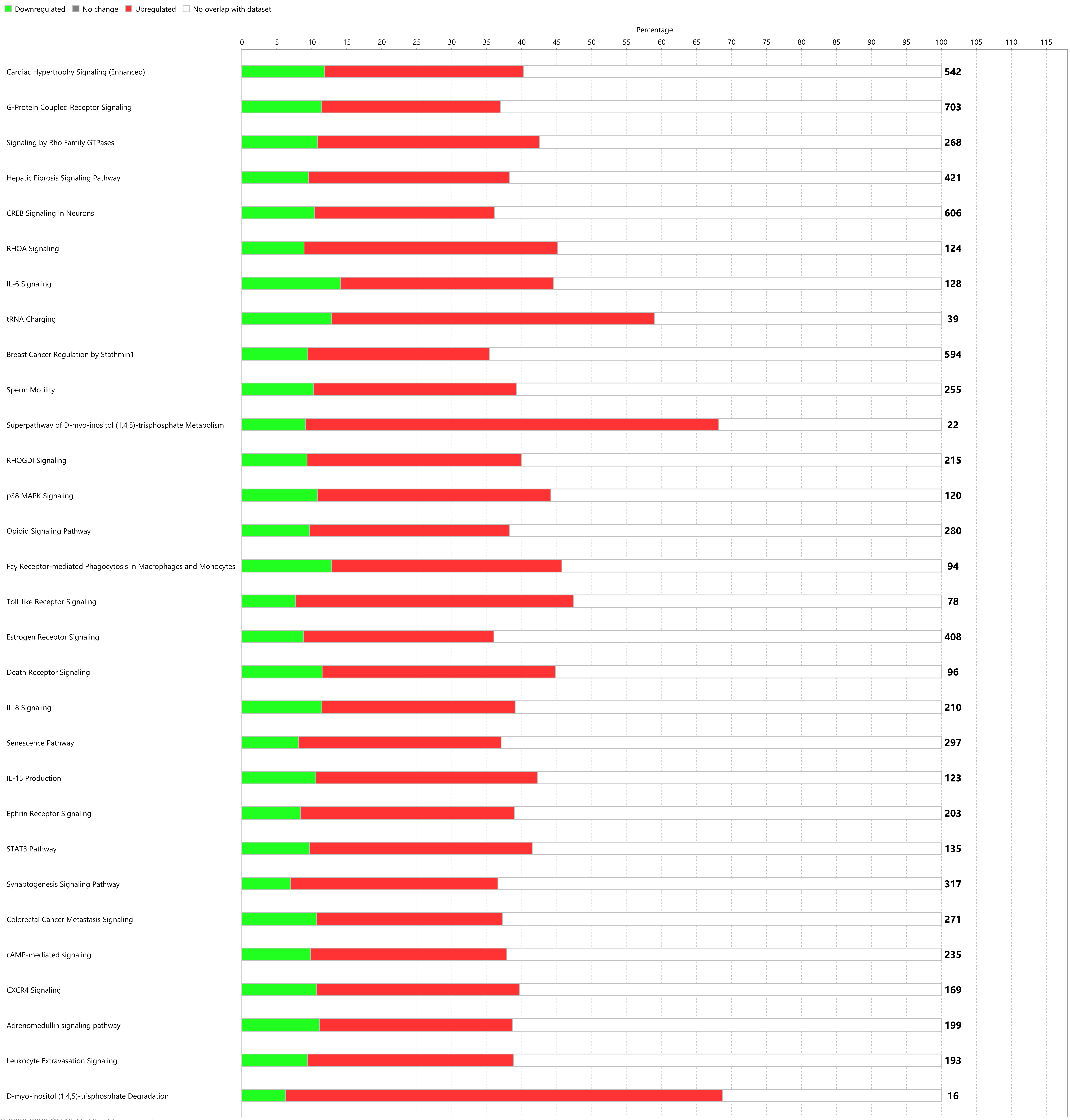

© 2000-2022 QIAGEN. All rights reserved.

Figure S6. The significantly enriched canonical pathways showing the percentage of driver (red) and antagonizing (green) genes in each pathway for AGECL vs IGF1b in Brahman population. Significantly enriched canonical pathways were identified using Benjamini-Hochberg pvalues <0.01. AGECL, age at first corpus; IGF1b, serum levels of insulin growth hormone measured in bulls).The left hand side is the enriched canonical pathway and the right hand side is the percentage of the driver and the antagonizing genes in the pathway

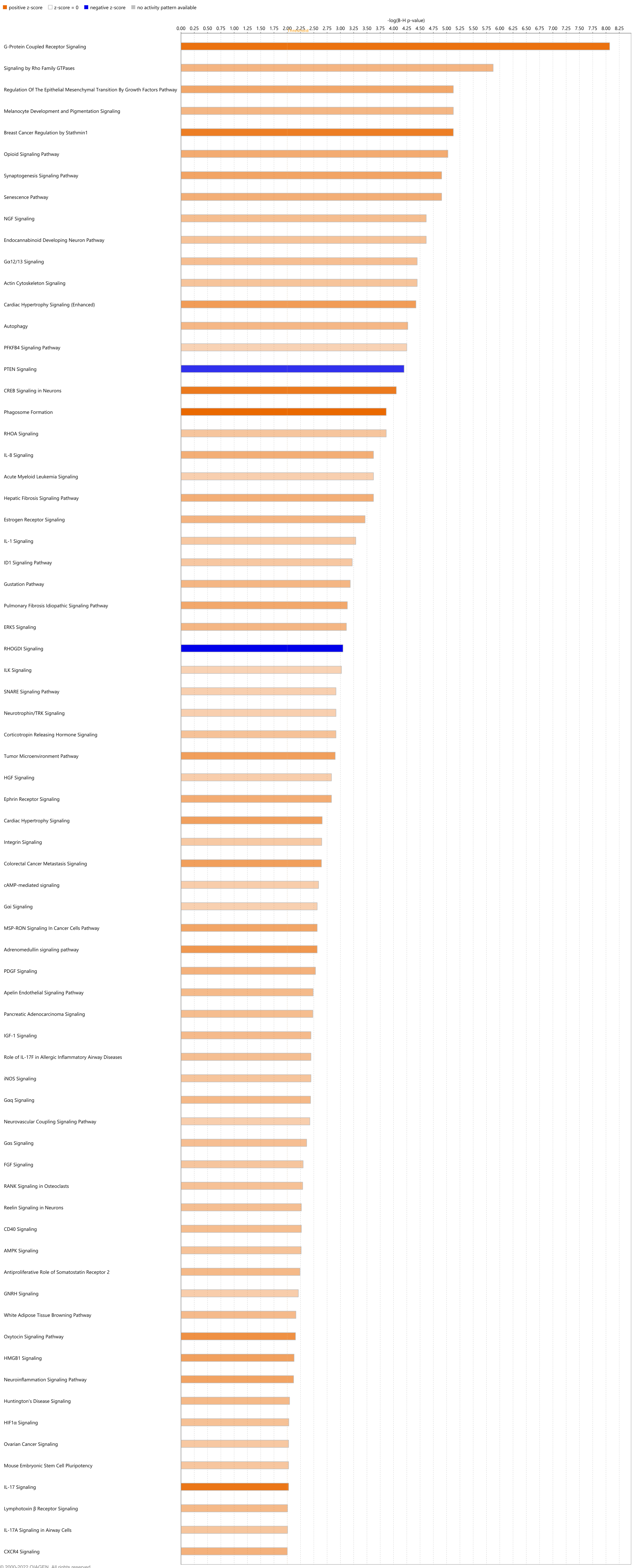

© 2000-2022 QIAGEN. All rights reserved.

Figure S7. Canonical pathways significantly enriched for AGECL vs IGF1b in Tropical Composite population. Significantly enriched canonical pathways were identified using Benjamini-Hochberg pvalues <0.01. AGECL, age at first corpus; IGF1b, serum levels of insulin growth hormone measured in bulls).The left hand side is the enriched canonical pathway and the right hand side is the predicted activation state of the canonical pathway using a z-score. Orange color indicate a positive z-score (Z>2) denoting the activation of the pathway. Blue color indicate a negative z-score (Z<-2) indicating the inhibition of the pathway.

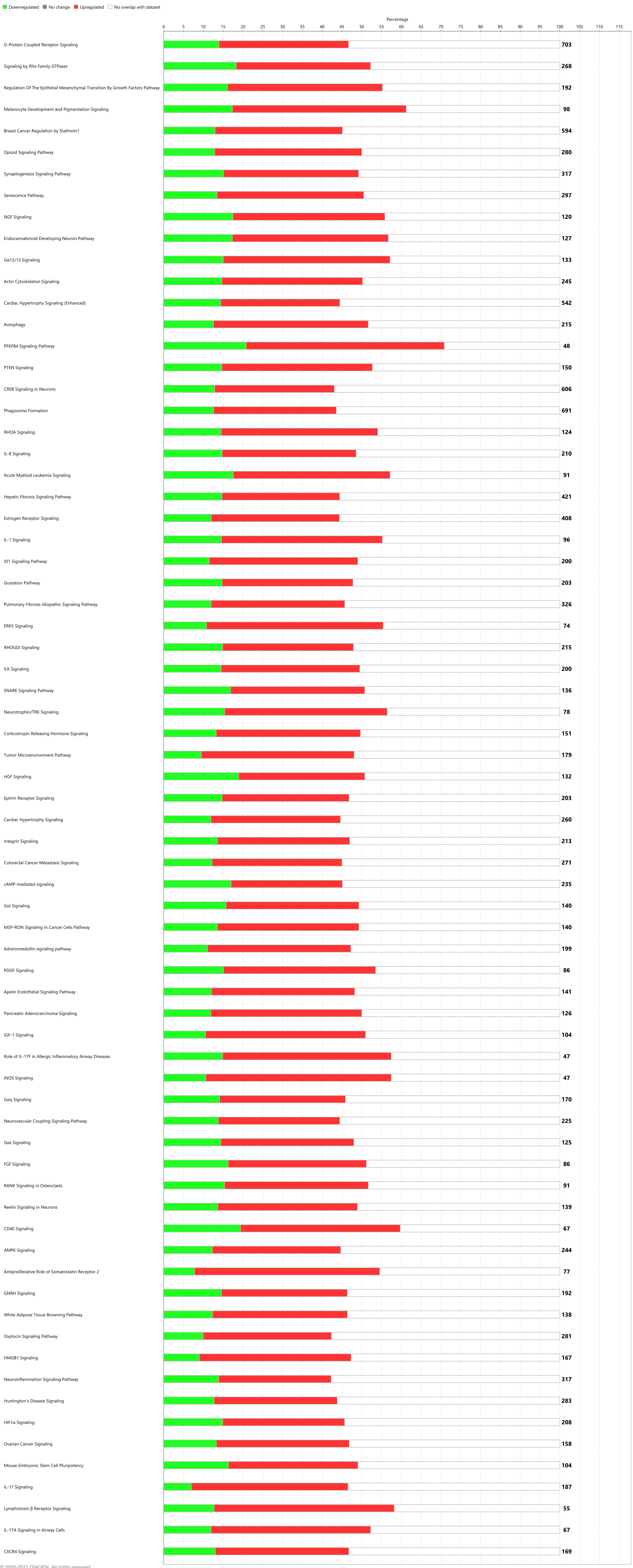

© 2000-2022 QIAGEN. All rights reserved.

Figure S8. The significantly enriched canonical pathways showing the percentage of driver (red) and antagonizing (green) genes in each pathway for AGECL vs IGF1b in Tropical Composite population. Significantly enriched canonical pathways were identified using Benjamini-Hochberg pvalues <0.01. AGECL, age at first corpus; IGF1b, serum levels of insulin growth hormone measured in bulls).The left hand side is the enriched canonical pathway and the right hand side is the percentage of the driver and the antagonizing genes in the pathway

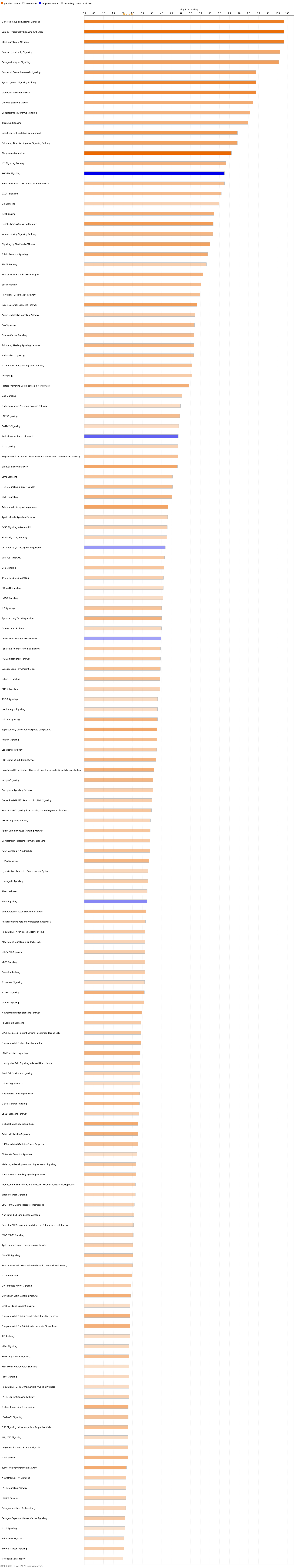

© 2000-2022 QIAGEN. All rights reserved.

Figure S9. Canonical pathways significantly enriched for IGF1c vs IGF1b in Brahman population. Significantly enriched canonical pathways were identified using Benjamini-Hochberg pvalues <0.01. IGF1, serum levels of insulin growth hormone (measured in bulls, IGF1b, or cows, IGF1c).The left hand side is the enriched canonical pathway and the right hand side is the predicted activation state of the canonical pathway using a z-score. Orange color indicate a positive z-score (Z>2) denoting the activation of the pathway. Blue color indicate a negative z-score (Z<-2) indicating the inhibition of the pathway.

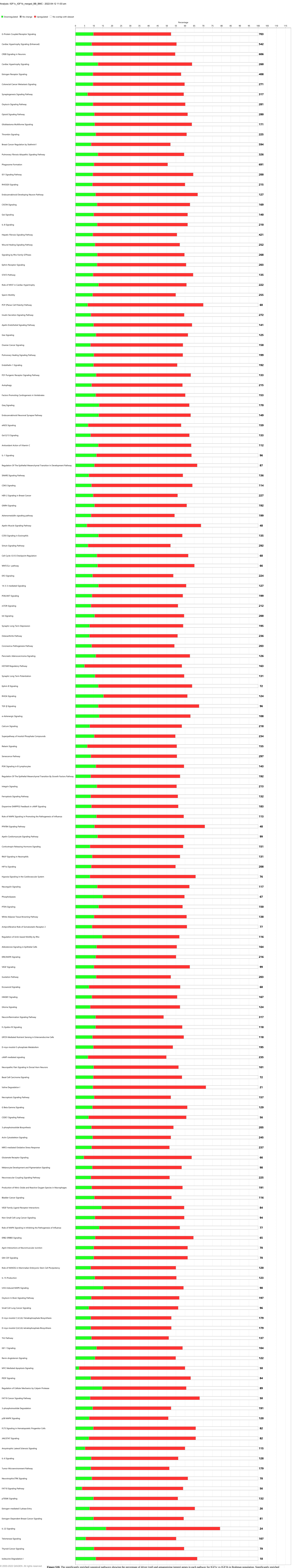

© 2000-2022 QIAGEN. All rights reserved.

Figure S10. The significantly enriched canonical pathways showing the percentage of driver (red) and antagonizing (green) genes in each pathway for IGF1c vs IGF1b in Brahman population. Significantly enriched canonical pathways were identified using Benjamini-Hochberg pvalues <0.01. IGF1, serum levels of insulin growth hormone (measured in bulls, IGF1b, or cows, IGF1c).The left hand side is the enriched canonical pathway and the right hand side is the percentage of the driver and the antagonizing genes in the pathway

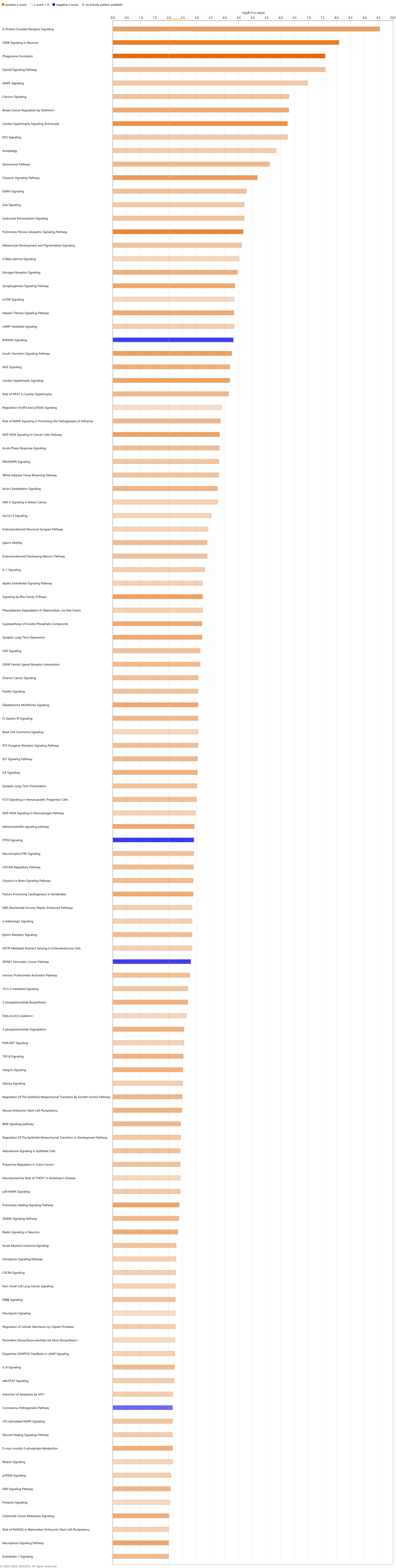

© 2000-2022 QIAGEN. All rights reserved.

Figure S11. Canonical pathways significantly enriched for IGF1c vs IGF1b in Tropical Composite population. Significantly enriched canonical pathways were identified using Benjamini-Hochberg pvalues <0.01. IGF1, serum levels of insulin growth hormone (measured in bulls, IGF1b, or cows, IGF1c).The left hand side is the enriched canonical pathway and the right hand side is the predicted activation state of the canonical pathway using a z-score. Orange color indicate a positive z-score (Z>2) denoting the activation of the pathway. Blue color indicate a negative z-score (Z<-2) indicating the inhibition of the pathway.

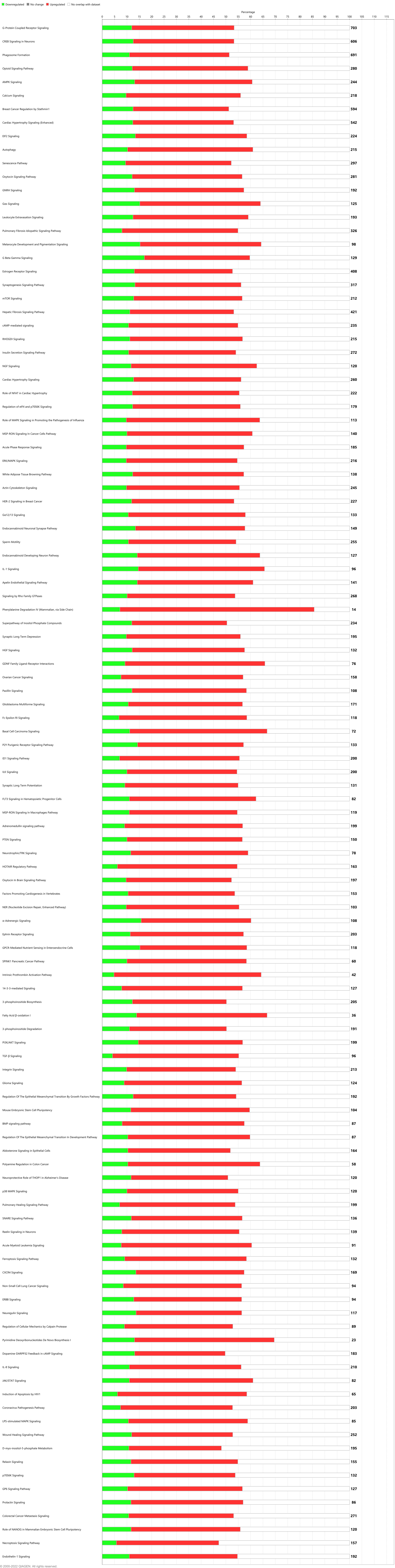

© 2000-2022 QIAGEN. All rights reserved.

Figure S12. The significantly enriched canonical pathways showing the percentage of driver (red) and antagonizing (green) genes in each pathway for IGF1c vs IGF1b in Tropical Composite population. Significantly enriched canonical pathways were identified using Benjamini-Hochberg pvalues <0.01. IGF1, serum levels of insulin growth hormone (measured in bulls, IGF1b, or cows, IGF1c).The left hand side is the enriched canonical pathway and the right hand side is the percentage of the driver and the antagonizing genes in the pathway
